# Gold-in-copper at low *CO coverage enables efficient electromethanation of CO_2_

**DOI:** 10.1038/s41467-021-23699-4

**Published:** 2021-06-07

**Authors:** Xue Wang, Pengfei Ou, Joshua Wicks, Yi Xie, Ying Wang, Jun Li, Jason Tam, Dan Ren, Jane Y. Howe, Ziyun Wang, Adnan Ozden, Y. Zou Finfrock, Yi Xu, Yuhang Li, Armin Sedighian Rasouli, Koen Bertens, Alexander H. Ip, Michael Graetzel, David Sinton, Edward H. Sargent

**Affiliations:** 1 Department of Electrical and Computer Engineering, University of Toronto, Toronto, ON Canada; 2 Department of Chemistry, The Chinese University of Hong Kong, Hong Kong, S.A.R. China; 3 Institute of Chemical Sciences and Engineering, École Polytechnique Fédérale de Lausanne, Lausanne, Switzerland; 4 Department of Materials Science and Engineering, University of Toronto, Toronto, ON Canada; 5 Department of Mechanical and Industrial Engineering, University of Toronto, Toronto, ON Canada; 6 Science Division, Canadian Light Source, Saskatoon, SK Canada; 7 Photon Science Division, Argonne National Laboratory, Lemont, IL USA

**Keywords:** Electrocatalysis, Energy, Nanoscale materials

## Abstract

The renewable-electricity-powered CO_2_ electroreduction reaction provides a promising means to store intermittent renewable energy in the form of valuable chemicals and dispatchable fuels. Renewable methane produced using CO_2_ electroreduction attracts interest due to the established global distribution network; however, present-day efficiencies and activities remain below those required for practical application. Here we exploit the fact that the suppression of *CO dimerization and hydrogen evolution promotes methane selectivity: we reason that the introduction of Au in Cu favors *CO protonation vs. C−C coupling under low *CO coverage and weakens the *H adsorption energy of the surface, leading to a reduction in hydrogen evolution. We construct experimentally a suite of Au-Cu catalysts and control *CO availability by regulating CO_2_ concentration and reaction rate. This strategy leads to a 1.6× improvement in the methane:H_2_ selectivity ratio compared to the best prior reports operating above 100 mA cm^−2^. We as a result achieve a CO_2_-to-methane Faradaic efficiency (FE) of (56 ± 2)% at a production rate of (112 ± 4) mA cm^−2^.

## Abstract (web summary)

The electroreduction of CO_2_ offers a promising approach to produce carbon-neutral methane using renewable electricity. This study shows that the introduction of Au in Cu enables selective methane production from CO_2_ by regulating *CO availability.

## Introduction

The CO_2_ electroreduction reaction (CO_2_RR) to valuable fuels and feedstocks, powered using renewable electricity, offers a sustainable approach to store intermittent renewable energy^1^. Prior CO_2_RR studies have reported the generation of C_1_ to C_3_ chemicals such as CO, methane, formate, ethylene, ethanol, and n-propanol^2–10^. Among these products, carbon-neutral methane produced from CO_2_RR is desired due to well-established natural gas infrastructure^11^.

Practical CO_2_RR systems need to produce a desired product with high selectivity, conversion rate, and energy efficiency (EE)^12,13^. In prior reports, most advances in improving the selectivity to methane in CO_2_RR operate at current densities below 50 mA cm^−2^ (ref. ^14–18^). Techno-economic analyses suggest that compelling CO_2_RR systems require current densities above 100 mA cm^−2^ (ref. ^19^), which prompted us to concentrate on improving the performance of CO_2_RR to methane in high current density regimes (>100 mA cm^−2^).

In CO_2_RR, *CO protonation to *CHO is the potential-determining step for methane formation, and it competes with C–C coupling toward C_2_ products^20,21^. In addition, *CO protonation competes with the hydrogen evolution reaction (HER), since both need *H (ref. ^22^). The simultaneous suppression of both HER and C–C coupling will improve methane selectivity.

Early studies by Hori et al.^2^ showed that Cu is the transition metal catalyst that generates methane and C_2+_ products; but that it did so with low product selectivity. Introducing a second metal into Cu has been shown to be a promising route to tune the product selectivity in CO_2_RR (refs. ^23–30^). Prior studies report that Au–Cu bimetallic catalysts of varying structures exhibit good selectivity to CO or alcohols, albeit with pure CO_2_ feeds (refs. ^31–34^). Here we present a strategy wherein we regulate *CO availability on Au–Cu catalysts, enabling selectivity to methane at high production rates in CO_2_RR. Density functional theory (DFT) calculations indicate that the introduction of Au in Cu not only steers the selectivity from C–C coupling to *CO protonation under low *CO coverage, but also tends to suppress HER relative to Cu. By implementing this concept experimentally, we achieve an FE of (56 ± 2)% to methane. The methane:H_2_ selectivity ratio is improved 1.6× compared with prior reports having a total current density above 100 mA cm^−2^ (Supplementary Table 1) (refs. ^35–39^).

## Results

### DFT calculations

In a previous study, we found that lowering the *CO coverage on a Cu surface improved the selectivity to methane in CO_2_RR while still suffering from prominent HER (ref. ^35^). Introducing a second element to Cu, such as Ag, has been shown to suppress HER (refs. ^21,28^). Au—like Ag—has a greater free energy of hydrogen adsorption than Cu, suggesting that it is also a poor HER catalyst^40^. We thus use Au–Cu as a representative example to assess methane selectivity on catalysts with HER-suppressing dopants under different *CO coverages. In addition, we note that selecting an element that is on the same side of the hydrogen adsorption volcano curve as Cu avoids any synergistic effects that may optimize the *H binding energy leading to better HER, such as with Cu–Ni or Cu–Pt (refs. ^41–43^).

**Fig. 1:**
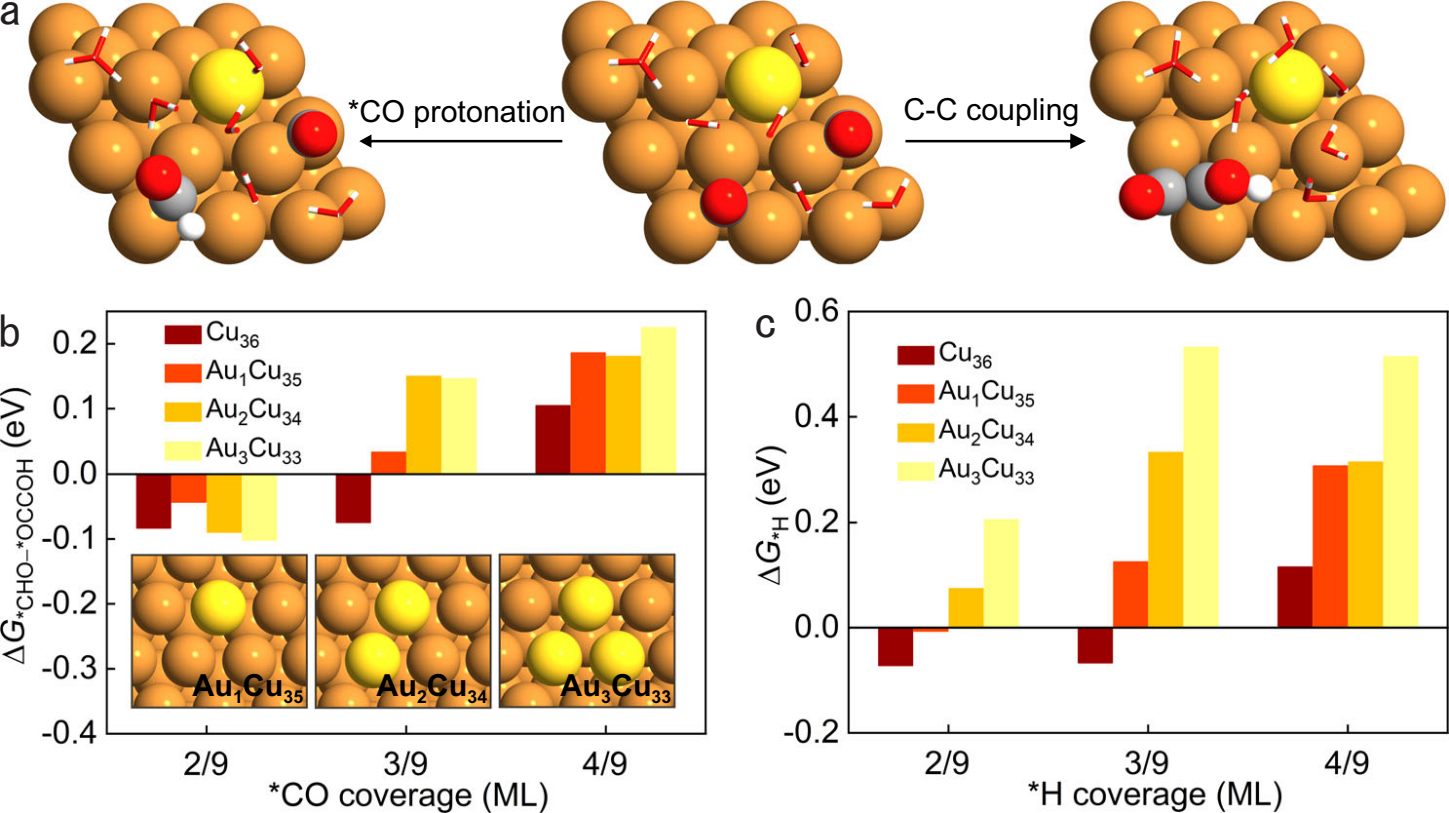
DFT studies. **a** Geometries of *CO, *CHO, and *OCCOH intermediates on Au–Cu surface, showing the competitive steps of *CO protonation and C–C coupling. **b** Reaction free energy difference between *CO protonation and C–C coupling steps on Cu_36_, Au_1_Cu_35_, Au_2_Cu_34_, and Au_3_Cu_33_ surfaces under varying *CO coverages. **c** Reaction free energies for *H intermediate formation under varying *H coverages on Cu_36_, Au_1_Cu_35_, Au_2_Cu_34_, and Au_3_Cu_33_ surfaces. Cu, Au, C, O, and H atoms in Fig. 1 are illustrated as orange, yellow, grey, red, and white spheres, respectively, while the water molecules are shown as sticks. Source data are provided as a Source Data file.

Computationally, we built three Au–Cu surfaces by replacing one, two, or three surface Cu atoms of a (3 × 3 × 4) Cu(111) supercell with Au atoms, denoted Au_1_Cu_35_, Au_2_Cu_34_, and Au_3_Cu_33_, respectively. With DFT, we first calculated the reaction free energies of *CO to *CHO (∆G_*CHO_) for methane formation and C–C coupling (∆G_*OCCOH_) for C_2_ products on these Au–Cu surfaces under different *CO coverages (Fig. 1a, Supplementary Figs. 1–4, and Supplementary Tables 2 and 3). ∆G_*CHO_–∆G_*OCCOH_ is used as a descriptor of the propensity for *CO protonation vs. C–C coupling. We found that the values of ∆G_*CHO_–∆G_*OCCOH_ on Au–Cu surfaces decrease when one reduces *CO coverage from 4/9 to 2/9 monolayer (ML) (Fig. 1b), a trend similar to that on Cu. Thus lowering *CO coverage on Au–Cu surfaces is predicted to favor methane vs. C_2_ products, as previously shown on Cu (ref. ^35^). In addition, DFT calculation results show that the values of ∆G_*CHO_–∆G_*OCCOH_ on Au–Cu surfaces are not always lower than the values of ∆G_*CHO_–∆G_*OCCOH_ on Cu at different *CO coverages (Fig. 1b), suggesting that only under low *CO coverage do some Au–Cu surfaces show a higher ratio of methane to C_2_ products compared to Cu.

To compare the HER activities on Cu and Au–Cu surfaces, we calculated the reaction free energies of *H intermediate formation (∆G_*H_) (Fig. 1c and Supplementary Fig. 5). The results suggest that Au–Cu surfaces tend to suppress HER compared to Cu under high *H coverages (Fig. 1c).

Taken together, these DFT studies suggest that Au–Cu tends to promote methane selectivity with low *CO coverage, since this will suppress C–C coupling; and that Au–Cu will further advance CO_2_RR over HER compared to pure Cu.

### Preparation and characterization of catalysts

**Fig. 2:**
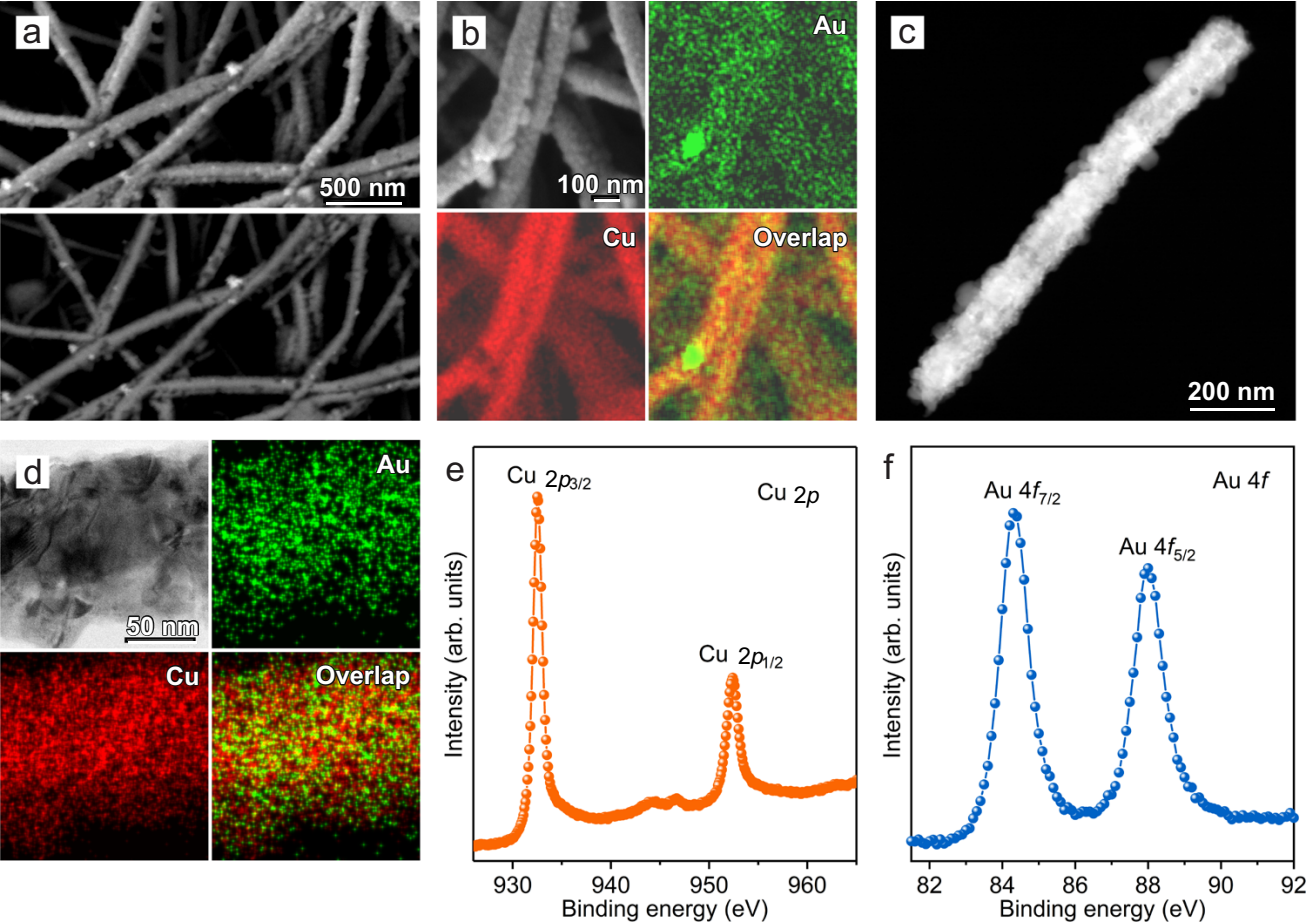
Structural and compositional analyses of 7% Au–Cu catalysts on PTFE. **a** Low magnification secondary electron image (above) and the corresponding backscattered electron image (below) of the 7% Au–Cu/PTFE, showing the dispersed Au nanoparticles (bright spots). **b** Secondary electron image and the corresponding EDX elemental mapping of Au and Cu for the 7% Au–Cu/PTFE. **c** High-angle annular dark-field scanning transmission electron microscopy (HAADF-STEM) image of one 7% Au–Cu/PTFE nanofiber. **d** High-magnification bright-field STEM image and the corresponding elemental mapping of Au and Cu from a section of one 7% Au–Cu/PTFE nanofiber. **e**, **f** High-resolution XPS spectra of Cu 2*p* (**e**) and Au 4*f* (**f**) for 7% Au–Cu/PTFE.

To achieve the goal of high selectivity to methane in CO_2_RR with high current densities, we sought to fabricate Au–Cu catalysts. We used a galvanic replacement approach enabled by the differing reduction potentials of Au and Cu (ref. ^44^). Firstly, we prepared a 100 nm thick layer of Cu catalysts on the surface of polytetrafluoroethylene (PTFE) nanofibers via sputter deposition (Supplementary Figs. 6 and 7). We then immersed the Cu/PTFE in an N_2_-saturated HAuCl_4_ aqueous solution at 65 °C for 15 min to prepare the Au–Cu catalysts on PTFE as the electrodes (Fig. 2a–d) via the galvanic replacement between Cu and AuCl_4_^−^—this approach allows us to directly tune the ratio of Au and Cu on PTFE substrates.

Low-magnification scanning electron microscope (SEM) images and energy-dispersive X-ray spectroscopy (EDX) elemental mapping show a uniform distribution of elemental Cu and Au on PTFE nanofibers, accompanied by loosely distributed Au nanoparticles also on the nanofibers (Fig. 2a, b). The bright-field scanning transmission electron microscope (STEM) image with higher magnification and corresponding EDX elemental mapping further confirms that Au and Cu are distributed evenly on the PTFE nanofibers (Fig. 2d). High-resolution X-ray photoelectron spectroscopy (XPS) characterization of Au–Cu electrodes shows the presence of Au^0^ and Cu which has been partially oxidized to Cu^+^ in the air (Fig. 2e, f and Supplementary Figs. 8 and 9)^45^. The atomic percentage of Au in the catalyst surface is approximately 7% determined by XPS (denoted 7% Au–Cu), which is lower than previously reported Au–Cu alloy catalysts studied in CO_2_RR (refs. ^31–33^).

### Investigation of CO_2_ electroreduction

**Fig. 3:**
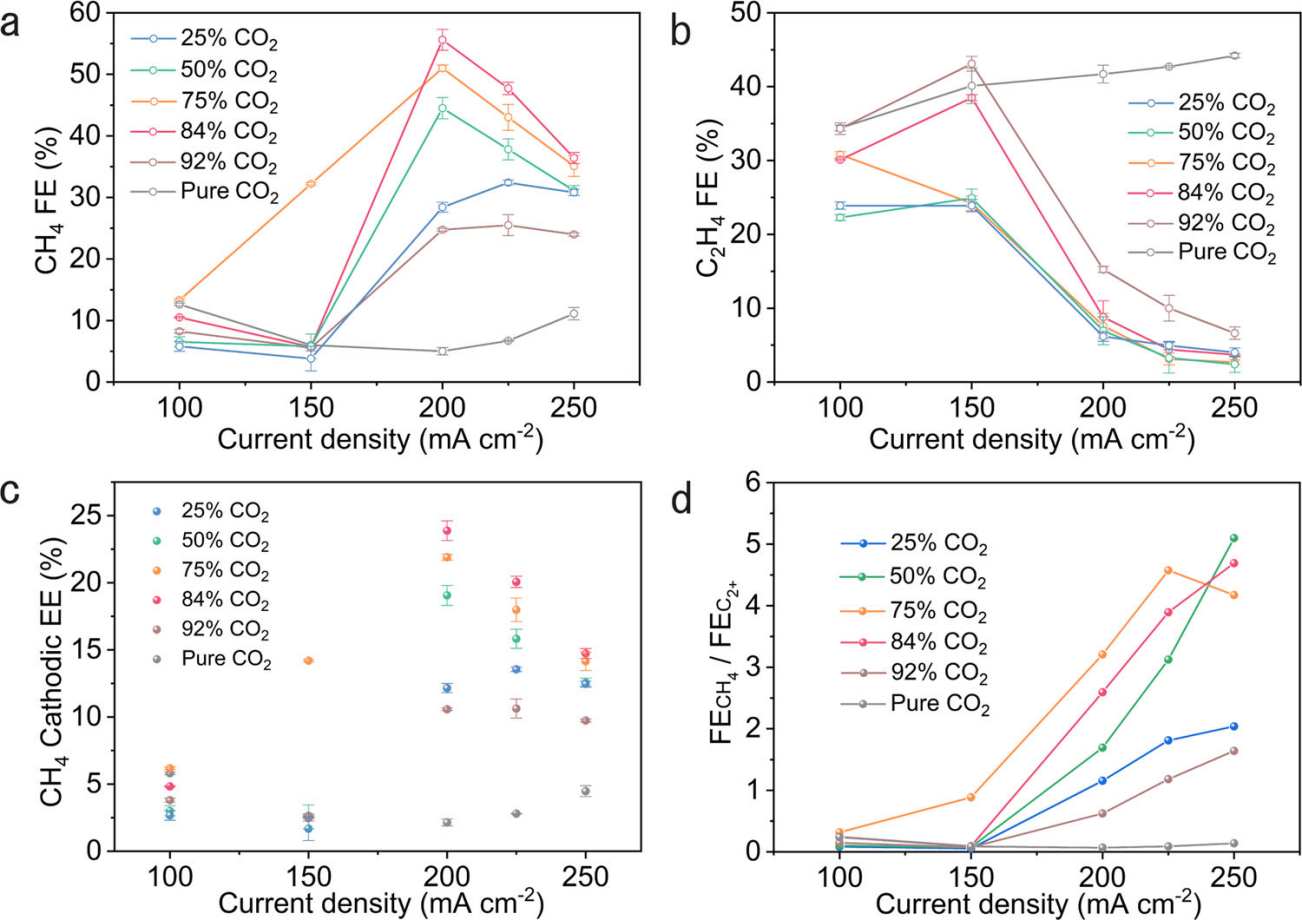
CO_2_RR performance of 7% Au–Cu catalysts at various CO_2_ concentrations. **a** Methane FEs on 7% Au–Cu at various CO_2_ concentrations. **b** Ethylene FEs on 7% Au–Cu at various CO_2_ concentrations. **c** CH_4_ cathodic EEs at different current densities under different CO_2_ concentrations. **d** Comparison of ${\rm{FE}}_{{\rm{CH}}_{4}}/{\rm{FE}}_{{\rm{C}}_{2+}}$ ratios on 7% Au–Cu catalysts at various CO_2_ concentrations. Error bars represent the standard deviation based on three separate measurements.

The CO_2_RR experiments were performed in a flow cell reactor with a three-electrode configuration (Supplementary Figs. 10 and 11) using CO_2_-saturated 1 M KHCO_3_ aqueous solution as the electrolyte. Previous studies show that, in CO_2_RR, both reaction rate—determined by current density—and CO_2_ concentration affect the concentration of *CO on the catalyst surface^35^. We thus evaluated the CO_2_RR performance of 7% Au–Cu electrodes by supplying gas streams consisting of different volume ratios of CO_2_ to N_2_ (Fig. 3, Supplementary Figs. 12 and 13, and Supplementary Table 4).

Figure 3a, b shows FEs of methane and ethylene on 7% Au–Cu catalysts in the current density range of 100–250 mA cm^−2^ at various CO_2_ concentrations (25% CO_2_, 50% CO_2_, 75% CO_2_, 84% CO_2_, 92% CO_2_, and pure CO_2_). At low current densities (≤150 mA cm^−2^), 7% Au–Cu delivers appreciable ethylene FEs under pure CO_2_ and CO_2_–N_2_ mixed streams (Fig. 3b). However, at high current densities (200–250 mA cm^−2^), relative to pure CO_2_, the methane FEs on 7% Au–Cu catalysts increase sharply in CO_2_–N_2_ mixed streams while the ethylene FEs decrease dramatically, which we ascribe to the low *CO coverage on catalyst surfaces as a result of the reduced CO_2_ concentration and high reaction rate. We note that, once they reach their peaks in these mixed streams, the methane FEs start to decrease with further increase in current density (Fig. 3a), a finding we attribute to the lack of *CO for the *CO protonation step of methane formation^46^. In particular, at 84% CO_2_, we achieve the highest CH_4_ FE of (56 ± 2)% on 7% Au–Cu catalysts with a CH_4_ production rate of (112 ± 4) mA cm^−2^. We calculated the CH_4_ cathodic EEs at different current densities under different CO_2_ concentrations (Fig. 3c): the highest CH_4_ cathodic EE of (24 ± 1)% was achieved at 200 mA cm^−2^ under 84% CO_2_.

To evaluate experimentally the selectivity between *CO protonation and C−C coupling reaction steps, we further calculated the ratios of methane FE to total C_2+_ FE (${{\rm{FE}}}_{{{{\rm{CH}}}}_{4}}/{{\rm{FE}}}_{{\rm{C}}_{2+}}$) on 7% Au–Cu catalysts at various CO_2_ concentrations (Fig. 3d). With low reaction rates (≤150 mA cm^−2^), the C_2+_ selectivity is higher than the methane selectivity regardless of the CO_2_ concentration. Under high current densities (200–250 mA cm^−2^), the ${\rm{FE}}_{{\rm{CH}}_{4}}/{\rm{FE}}_{{\rm{C}}_{2+}}$ ratio on 7% Au–Cu catalysts in CO_2_–N_2_ mixed streams is much greater than that in pure CO_2_, suggesting that low *CO coverage on the surface of Au–Cu catalysts—as a consequence of a reduced CO_2_ concentration and high current density—promotes the *CO protonation step for methane production, consistent with our DFT calculations.

**Fig. 4:**
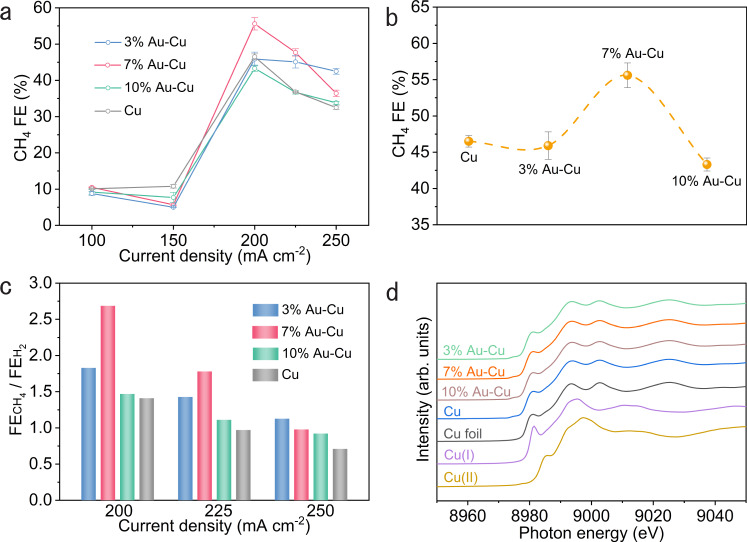
CO_2_RR performance at 84% CO_2_ and operando XAS characterization. **a** Methane FEs on different catalysts at 84% CO_2_. **b** Comparison of methane FEs on different catalysts at 200 mA cm^−2^. Error bars represent the standard deviation based on three separate measurements. **c** Comparison of ${\rm{FE}}_{{\rm{CH}}_{4}}/{\rm{FE}}_{{\rm{H}}_{2+}}$ ratios on different catalysts at 84% CO_2_. **d** Operando Cu K-edge XANES spectra of different catalysts during CO_2_RR with a constant current density of 200 mA cm^−2^. Bulk Cu foil, Cu_2_O, and CuO are listed as references.

To explore the effect of Au concentration on CO_2_RR performance under CO_2_–N_2_ co-feeds, we also prepared 3% Au–Cu and 10% Au–Cu catalysts on PTFE through a similar galvanic replacement approach (Supplementary Figs. 7 and 14–19) and measured CO_2_RR performance of the 3% Au–Cu, 10% Au–Cu, and Cu catalysts at 84% CO_2_ for comparison (Fig. 4a–c, Supplementary Figs. 20 and 21, and Supplementary Table 5). At low reaction rates (≤150 mA cm^−2^), the methane FEs on 3% Au–Cu, 7% Au–Cu, 10% Au–Cu, and Cu catalysts are below 11% (Fig. 4a), while ethylene and ethanol are the main CO_2_RR products on these catalysts (Supplementary Fig. 20c and Supplementary Table 5). At high reaction rates (200–250 mA cm^−2^), methane becomes the main CO_2_RR product while ethylene FEs are below 12% on the 3% Au–Cu, 10% Au–Cu, and Cu catalysts; methane FEs on 3% Au–Cu, 10% Au–Cu, and Cu catalysts give peak values at 200 mA cm^−2^ and then decrease along with the increase in current density (Fig. 4a). These trends are similar to that observed on the 7% Au–Cu catalysts. By comparing the highest methane FEs on different catalysts, we note that, among the catalysts studied, only 7% Au–Cu catalysts deliver higher methane FE vs. Cu catalysts (Fig. 4b), suggesting the significance of controlling Au concentration in Au–Cu catalysts for promoting methane selectivity. The 7% Au–Cu delivers—compared to 3% Au–Cu and 10% Au–Cu—higher methane FE at 200 mA cm^−2^. We associate this with improved suppression of HER on 7% Au–Cu (Supplementary Fig. 20a) and note that both HER and *CO coverage impact methane FE. We also calculated the ${\rm{FE}}_{{\rm{CH}}_{4}}/{\rm{FE}}_{{\rm{C}}_{2+}}$ ratios on Cu and three Au–Cu catalysts at 84% CO_2_: only at high current density—low *CO coverage—do some of Au–Cu catalysts show higher ${{\rm{FE}}}_{{{{\rm{CH}}}}_{4}}/{{\rm{FE}}}_{{\rm{C}}_{2+}}$ ratios compared to Cu, in agreement with DFT calculations.

At high current densities (200–250 mA cm^−2^) with high methane selectivity, the H_2_ FEs on 3% Au–Cu, 7% Au–Cu, and 10% Au–Cu catalysts are lower than that on the Cu catalysts (Supplementary Fig. 20a), suggesting that the introduction of Au in Cu tends to suppress HER when using dilute CO_2_ feeds. We further calculated the ratio of methane FE to H_2_ FE (${\rm{FE}}_{{\rm{CH}}_{4}}/{\rm{FE}}_{{\rm{H}}_{2}}$) on catalysts in high current density regimes (Fig. 4c): compared with Cu catalysts, 3% Au–Cu, 7% Au–Cu, and 10% Au–Cu catalysts exhibit higher ${\rm{FE}}_{{\rm{CH}}_{4}}/{\rm{FE}}_{{\rm{H}}_{2}}$ ratios—with the highest value of 2.7 on 7% Au–Cu catalysts— indicating that the Au–Cu catalysts shifted the reaction from undesired HER toward *CO protonation for methane production.

To investigate the chemical state of Cu in the catalysts during CO_2_RR, we carried out operando X-ray absorption spectroscopy (XAS) at the Cu K-edge at a constant current density of 200 mA cm^−2^ with an 84% CO_2_ feed (Fig. 4d). The average valence states of Cu in 3% Au–Cu, 7% Au–Cu, 10% Au–Cu, and Cu catalysts are zero during CO_2_RR, demonstrating that the difference in product selectivity among these catalysts is associated with the metallic state of Cu in lieu of copper oxides^5,47^.

## Discussion

This work demonstrates that the introduction of Au in Cu facilitates *CO protonation for methane formation using CO_2_–N_2_ co-feeds and suppresses HER at high current densities. DFT results show that a decrease in *CO coverage on Au–Cu surfaces favors *CO protonation vs. C–C coupling; compared with Cu, Au–Cu suppresses HER, enabling methane selectivity improvements under dilute CO_2_ streams. Experimentally, we fabricated Au–Cu catalysts and regulated *CO availability by controlling the CO_2_ concentration and current density, wherein the selectivity ratio of methane to H_2_ exhibited the highest value of 2.7. We report a CO_2_-to-methane conversion with a high methane FE of (56 ± 2)% at a partial current density of (112 ± 4) mA cm^−2^ with a CO_2_–N_2_ co-feed. These findings suggest a promising strategy to convert CO_2_ to carbon-neutral methane with a combination of high selectivity, high conversion rate, and high cathodic EE through catalyst design and tuning local *CO coverage.

## Methods

### DFT calculations

In the Vienna ab initio simulation package, the generalized gradient approximation and the Perdew–Burke–Ernzerhof exchange-correlation functional was implemented for all DFT calculations^48–52^. The projector-augmented wave (PAW) method was used to treat the electron–ion interactions^53,54^ with an energy cut-off of 450 eV for the plane-wave basis set. The force and energy convergence for all DFT calculations were set to 0.01 eV Å^−1^ and 10^−5^ eV, respectively. A (3 × 3 × 4) Cu(111) supercell with the bottom two layers fixed was used to simulate the exposed Cu surface with a 15 Å vacuum gap. One, two, or three surface Cu atoms were substituted by Au atoms in the Au_1_Cu_35_, Au_2_Cu_34_, and Au_3_Cu_33_, respectively. A (3 × 3 × 1) Monkhorst–Pack *k*-points grid was used to optimize all the surface structures. In DFT calculations, we did not consider the isolated arrangement of Au dopants in Cu as the XAS characterization of the Au–Cu catalysts showed that Au atoms were not atomically dispersed in Cu (Supplementary Fig. 22 and Supplementary Table 6).

Surface *CO coverages of 2/9 ML, 3/9 ML, and 4/9 ML were studied, where 2/9 ML corresponds to two single-carbon adsorbed species or one double-carbon species on the surface of the supercell. To systematically determine the most stable geometry of each reaction intermediate in CO_2_RR and HER under different *CO coverages (2/9, 3/9, and 4/9 ML) on different surfaces (Cu_36_, Au_1_Cu_35_, Au_2_Cu_34_, and Au_3_Cu_33_), we considered different possibilities of *CO adsorption, protonation, and C–C coupling, as well as different directions of *OCCO protonation (more details of our computational workflow in Supplementary Fig. 23). We note that the models reported in this study include a charged water layer, i.e., an ML of six water molecules, one of which is a hydronium or charged water (H_3_O^+^) molecule, to consider both field and solvation effects^55^. The water structure was determined by ab initio molecular dynamics and adopted from a previous study^56^. The two competing CO_2_RR reaction steps as listed below^57–61^ were simulated for the three *CO coverages while *H adsorption was simulated for equivalent *H coverages

1 $$\ast {\rm{CO}}+{\rm{H}}_{2}{\rm{O}}+{\rm{e}}^{-}\to \ast {\rm{CHO}}+{\rm{OH}}^{-}$$

2 $$\ast {\rm{CO}}+\ast {\rm{CO}}+{{\rm{H}}}_{2}{\rm{O}}+{{\rm{e}}}^{-}\to \ast {\rm{OCCOH}}+{{\rm{OH}}}^{-}$$

3 $${{\rm{H}}}^{+}+{{\rm{e}}}^{-}+\ast \to \ast {\rm{H}}$$

The Gibbs free energy changes (∆*G*) for *CO protonation, C–C coupling, and *H adsorption were calculated without dipole corrections based on the computational hydrogen electrode (CHE) model^62^. The Gibbs free energy of adsorbed and non-adsorbed species (*G*) is calculated as

4 $$G=E+{\rm{ZPE}}+\smallint C{p}{\rm{d}}T-TS$$

where *E*, ZPE, *Cp*, and *S* are the electronic energy directly obtained from DFT calculations, zero-point energy, heat capacity, and entropy, respectively (see Supplementary Table 2 for more details). *T* is set to room temperature (298.15 K) for a better comparison with the experimental measurements.

### Electrode preparation

All chemicals were used as received without further purification. All aqueous solutions were prepared using deionized water with a resistivity of 18.2 MΩ cm. The Cu/PTFE cathodes were prepared by sputtering 100 nm thickness of Cu catalysts (Cu target, 99.999%, Kurt J. Lesker company) on PTFE membranes (pore size: 450 nm, Beijing Zhongxingweiye Instrument Co., Ltd.) using a magnetron sputtering system. In the cathode, a porous PTFE membrane functions as a stable hydrophobic gas diffusion layer and prevents flooding during operation^7^. 3% Au–Cu, 7% Au–Cu, and 10% Au–Cu cathodes were prepared by immersing the Cu/PTFE electrodes in N_2_-saturated HAuCl_4_ aqueous solution (5 μmol L^−1^) at 40 °C for 30 min, 65 °C for 15 min, and 65 °C for 30 min, respectively. Ag/AgCl reference electrode (3 M KCl, BASi) and Ni foam (1.6 mm thickness, MTI Corporation) was used as the reference electrode and anode, respectively. Ni foam was used as an OER electrode in the anode due to its commercial availability and good stability^35,63^.

Material characterization. SEM images and the corresponding EDX elemental mapping were taken using the Hitachi FE-SEM SU5000 microscope. HAADF-STEM and bright-field STEM images, and the corresponding EDX elemental mapping were taken using a Hitachi HF-3300 microscope at 300 kV. XRD was recorded on Rigaku SmartLab X-ray diffractometer with Cu-K*α* radiation. The surface compositions of electrodes were determined by XPS (Thermo Scientific K-Alpha) using a monochromatic aluminum X-ray source. Operando Cu K-edge XAS spectra recorded in fluorescence yield were performed at the SuperXAS beamline at the Swiss Light Source. Ex situ XAS measurements were carried out at the Advanced Photon Source (Argonne National Laboratory). XAS data were processed by Athena and Artemis software included in a standard IFEFFIT package^64^.

### Electrochemical measurements

The electrochemical measurements were conducted in an electrochemical flow cell setup configuration with the three-electrode system at an electrochemical station (AUT50783). The geometric area of the cathode in the flow cell is 1 cm^2^, which is used for all current density calculations. 30 mL of CO_2_-saturated 1 M KHCO_3_ aqueous solution was introduced into the cathode chamber and the anode chamber at the rate of 10 mL min^−1^ by two pumps, respectively. An anion exchange membrane (Fumasep FAB-PK-130, Fuel Cell Store) was used to separate the cathode chamber and anode chamber. Pure CO_2_ gas (Linde, 99.99%) or N_2_-diluted CO_2_ gas with different CO_2_ concentrations (75% and 84%) was continuously supplied to the gas chamber of the flow cell at a flow rate of 90 mL min^−1^. The CO_2_RR performance was tested using constant-current electrolysis while purging CO_2_ into the catholyte during the whole electrochemical test. The potentials vs. Ag/AgCl reference electrode were converted to values vs. reversible hydrogen electrode using the equation

5 $${E}_{\rm{RHE}}={E}_{{\rm{Ag}}/{\rm{AgCl}}}+0.210\,{\rm{V}}+0.0591\times {\rm{pH}}$$

The ohmic loss between the working and reference electrodes was evaluated by electrochemical impedance spectroscopy technique and 80% *i*R compensation was applied to correct the potentials manually.

Gas products were analyzed using a gas chromatograph (PerkinElmer Clarus 600) equipped with thermal conductivity and flame ionization detectors. Liquid products were analyzed by nuclear magnetic resonance spectrometer (Agilent DD2 600 MHz) and dimethylsulfoxide was used as an internal standard.

We calculated the methane cathodic EE based on the equation as follows^7^:

6 $${\rm{Cathodic}}\,EE=\frac{(1.23+(-{E}_{{\rm{methane}}}))\times F{E}_{{\rm{methane}}}}{(1.23+(-{E}_{{\rm{applied}}}))},$$

where the overpotential of oxygen evolution is assumed to be 0, *E*_applied_ is the potential used in the experiment, *FE*_methane_ is the measured Faradaic efficiency of methane in percentage, and *E*_methane_ = 0.17 V_RHE_ for CO_2_RR (ref. ^65^).

## Supplementary information

Supplementary Information

## Data Availability

The data supporting this study are available within the paper and the Supplementary Information. The source data of the geometries optimized by DFT calculations are provided in this paper. Source data are provided with this paper.

## Source data

Source Data
