# Supplementary material for: Gold-in-copper at low *CO coverage enables efficient electromethanation of CO2
Source: Nat Commun. 2021 Jun 7;12:3387. doi: 10.1038/s41467-021-23699-4 (PMC8184940; doi:10.1038/s41467-021-23699-4)
Supplement: Supplementary file 1 — Supplementary Information [file 41467_2021_23699_MOESM1_ESM.pdf]

Supplementary information

Gold-in-copper at low *CO coverage enables efficient electromethanation of CO_2

Xue Wang[1†], Pengfei Ou[1†], Joshua Wicks[1†], Yi Xie[2†], Ying Wang[2†], Jun Li[3], Jason Tam[4], Dan Ren[3], Jane Y. Howe[4], Ziyun Wang[1], Adnan Ozden[5], Y. Zou Finfrock[6,7], Yi Xu[5], Yuhang Li[1], Armin Sedighian Rasouli[1], Koen Bertens[1], Alexander H. Ip[1], Michael Graetzel[3], David Sinton[5], Edward H. Sargent[1]*

[1]*Department of Electrical and Computer Engineering, University of Toronto, Toronto, ON, Canada.*

[2]*Department of Chemistry, The Chinese University of Hong Kong, Hong Kong S. A. R., China.*

[3]*Institute of Chemical Sciences and Engineering, École polytechnique fédérale de Lausanne, Lausanne 1015, Switzerland.*

[4]*Department of Materials Science and Engineering, University of Toronto, Toronto, ON, Canada.*

[5]*Department of Mechanical and Industrial Engineering, University of Toronto, Toronto, ON, Canada.*

[6]*Science Division, Canadian Light Source, Saskatoon, SK, Canada.*

[7]*Photon Science Division, Argonne National Laboratory, Lemont, IL, USA.*

[†]These authors contributed equally to this work.

*Correspondence and requests for materials should be addressed to Edward H. Sargent (ted.sargent@utoronto.ca) (E.H.S.)

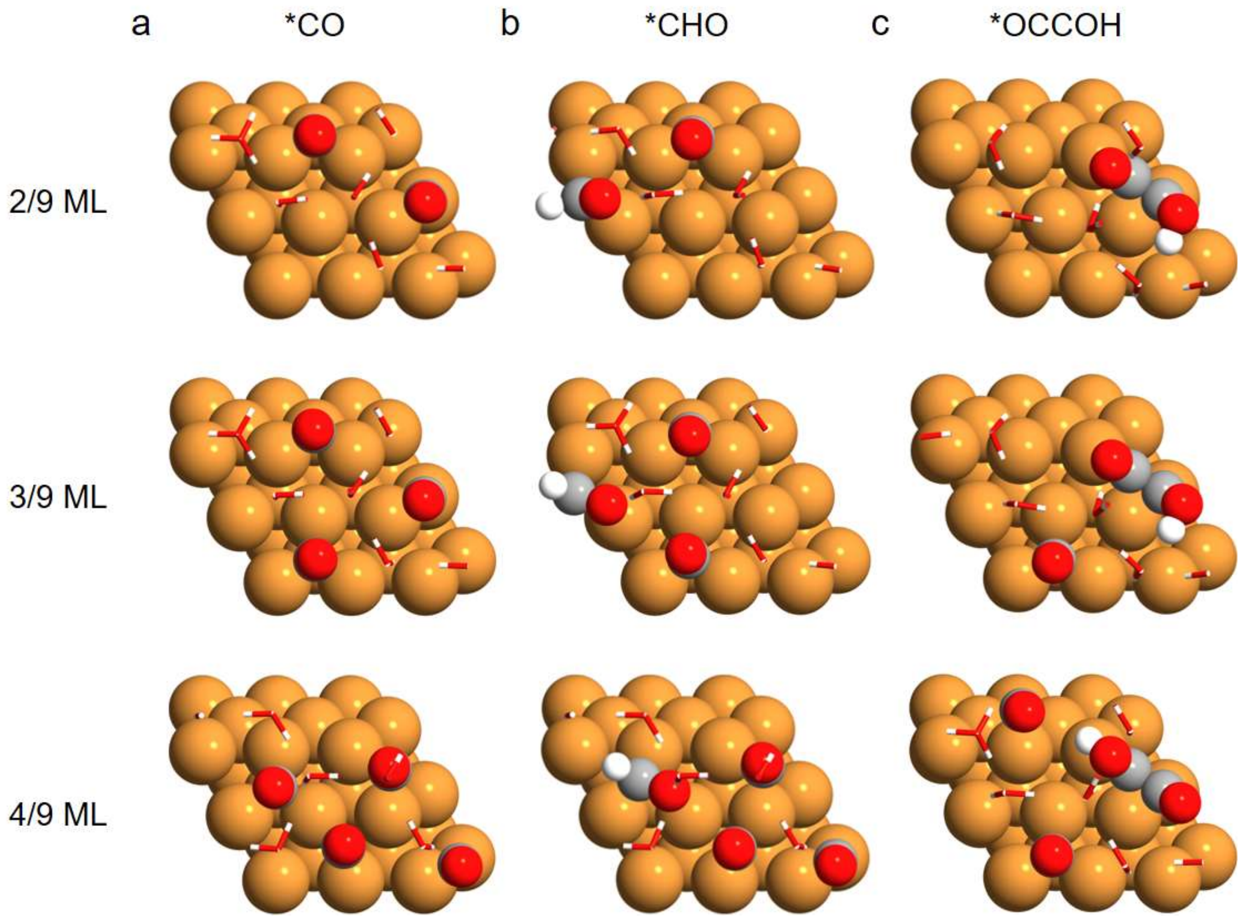

Supplementary Figure 1 | Geometries of *CO, *CHO, and *OCCOH on the Cu_{36} surface under different *CO coverages (2/9, 3/9, and 4/9 ML). a-c, Top views of *CO (**a**), *CHO (**b**), and *OCCOH (**c**). Orange, red, grey, and white spheres represent copper, oxygen, carbon, and hydrogen atoms, respectively. Water molecules are shown as sticks. These notations are used throughout the Supplementary Information. Source data are provided as a Source Data file.

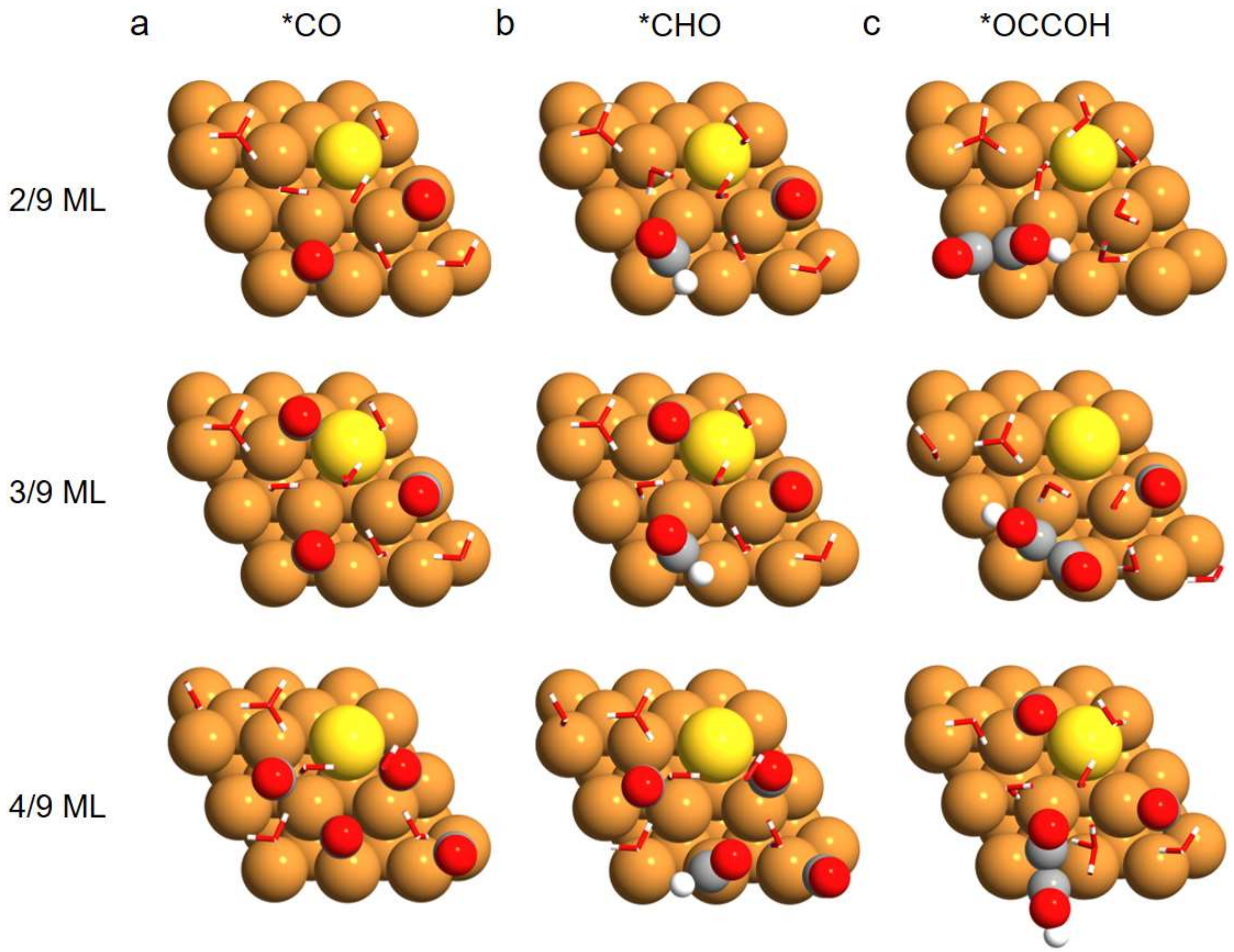

Supplementary Figure 2 | Geometries of *CO, *CHO, and *OCCOH on the Au_1Cu_{35} surface under different *CO coverages (2/9, 3/9, and 4/9 ML). a-c, Top views of *CO (**a**), *CHO (**b**), and *OCCOH (**c**). Yellow spheres represent gold. This notation is used throughout the Supplementary Information. Source data are provided as a Source Data file.

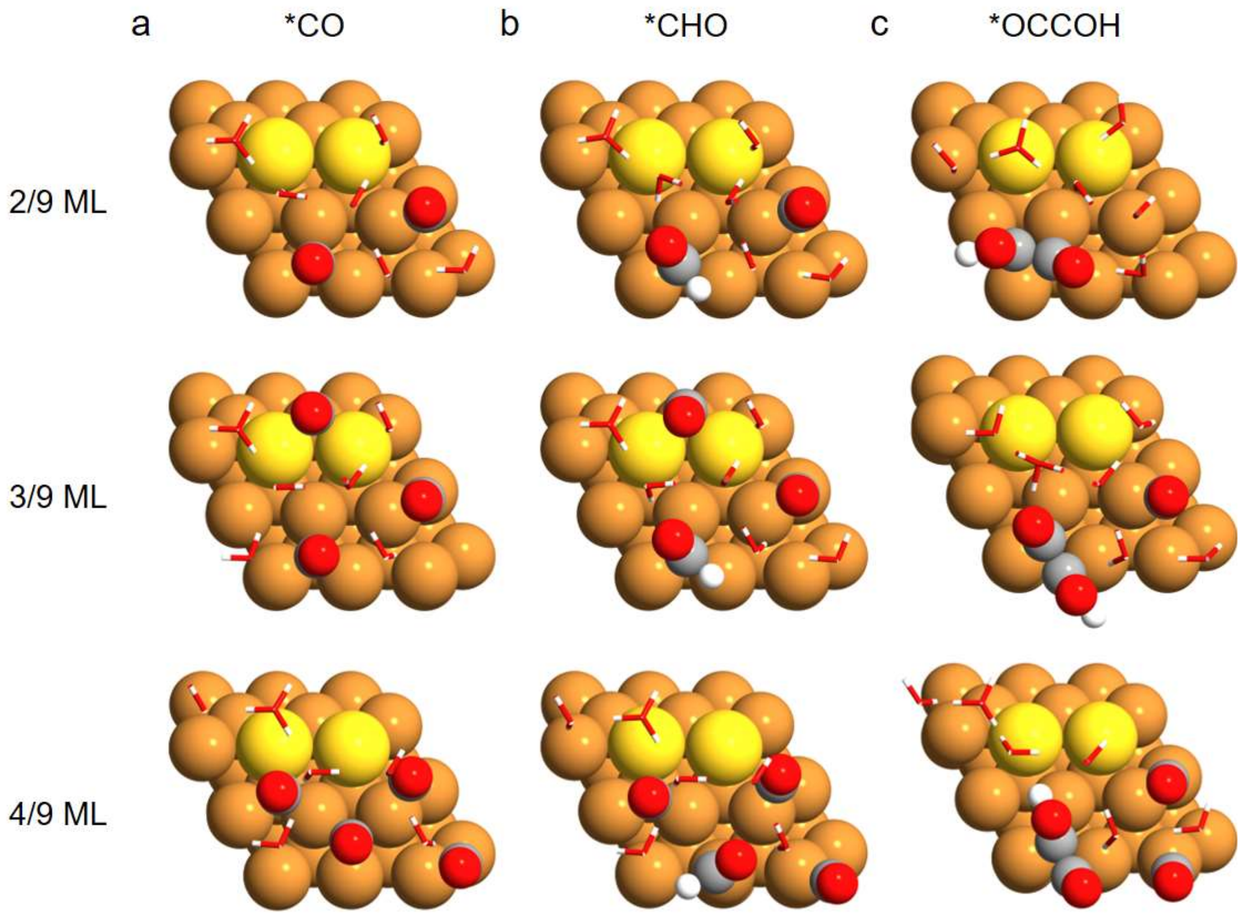

Supplementary Figure 3 | Geometries of *CO, *CHO, and *OCCOH on the Au_2Cu_{34} surface under different *CO coverages (2/9, 3/9, and 4/9 ML). a-c, Top views of *CO (**a**), *CHO (**b**), and *OCCOH (**c**). Source data are provided as a Source Data file.

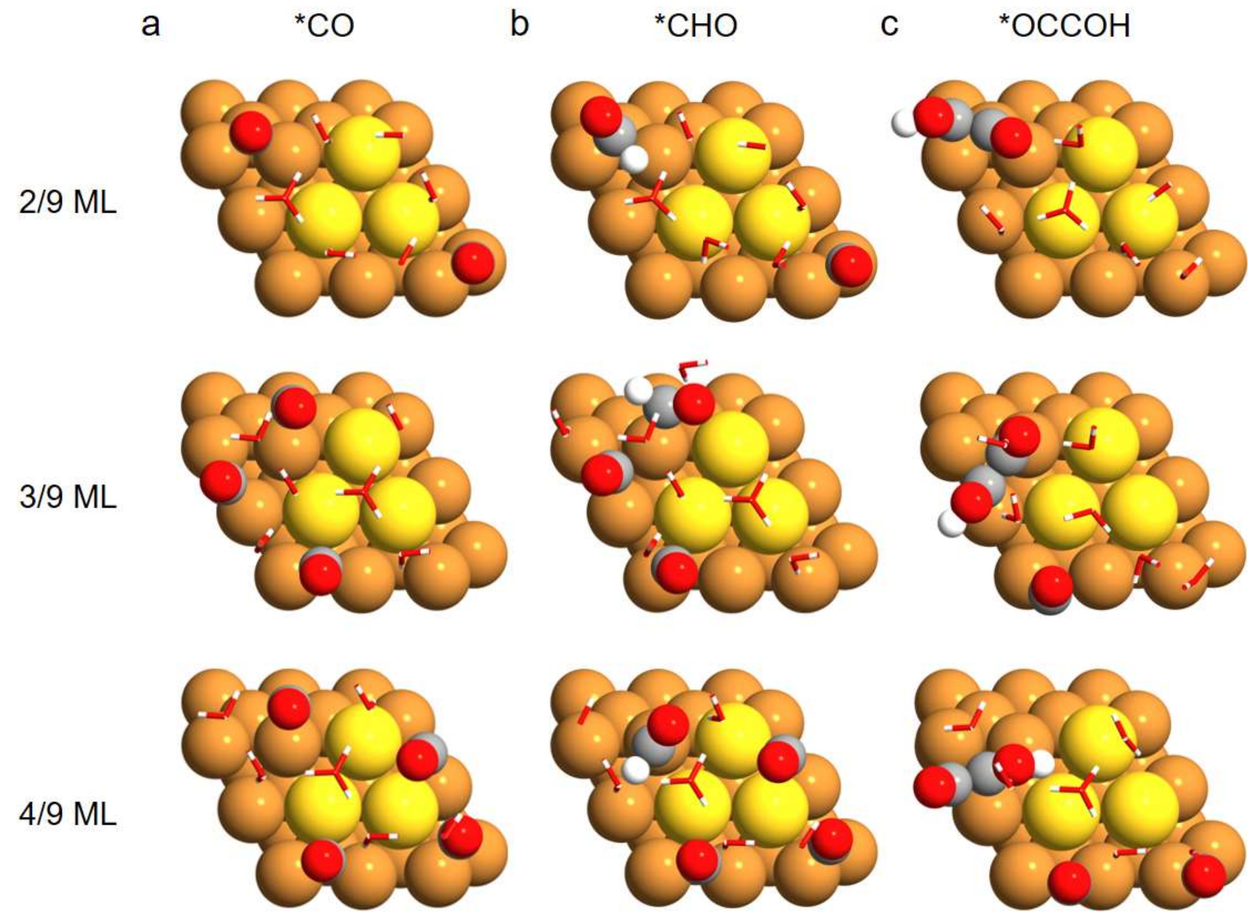

Supplementary Figure 4 | Geometries of *CO, *CHO, and *OCCOH on the Au_3Cu_{33} surface under different *CO coverages (2/9, 3/9, and 4/9 ML). a-c, Top views of *CO (**a**), *CHO (**b**), and *OCCOH (**c**). Source data are provided as a Source Data file.

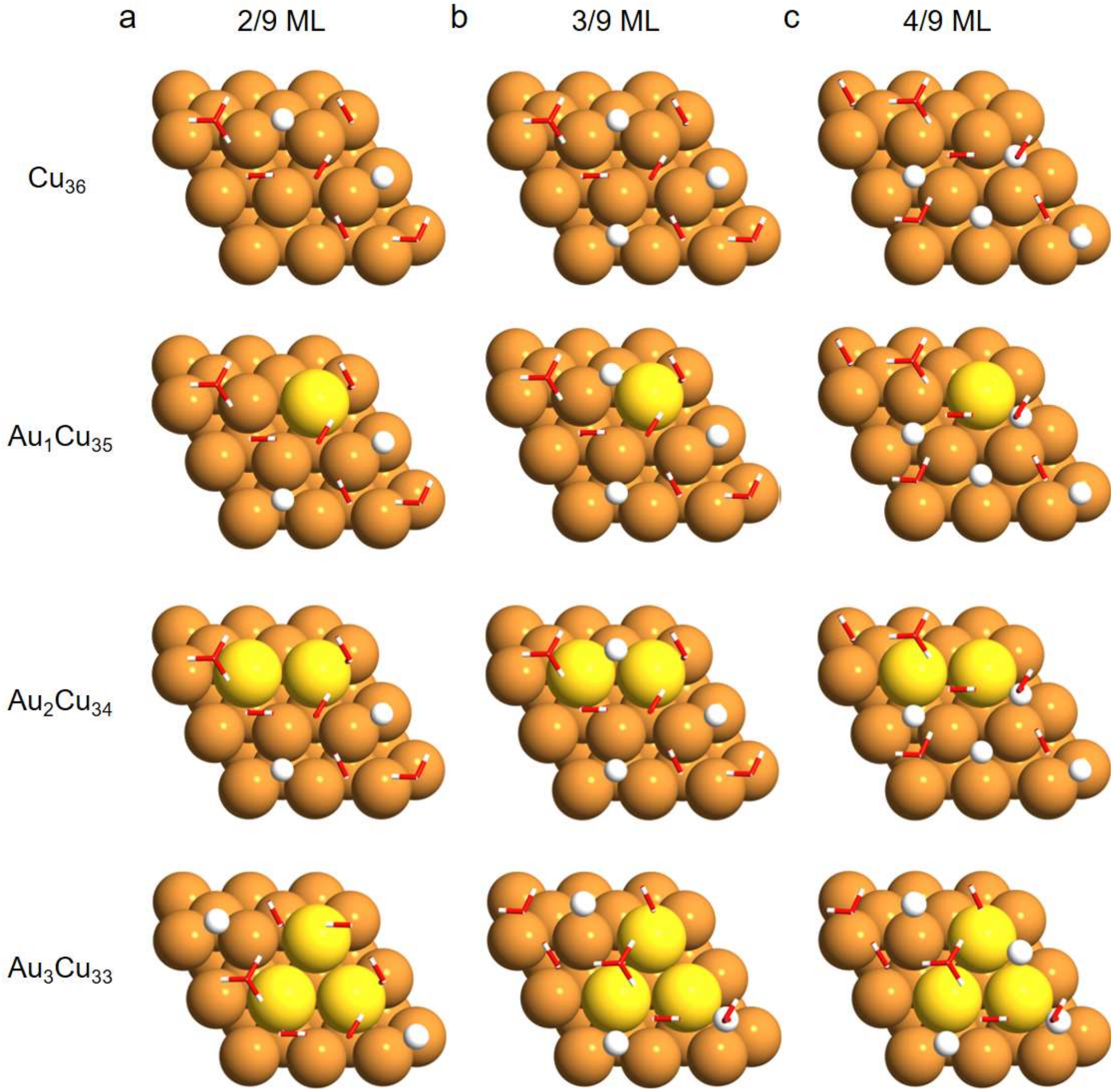

Supplementary Figure 5 | Top views of different coverages of *H intermediates on various surfaces. a, 2/9 ML. **b,** 3/9 ML. **c,** 4/9 ML. Source data are provided as a Source Data file.

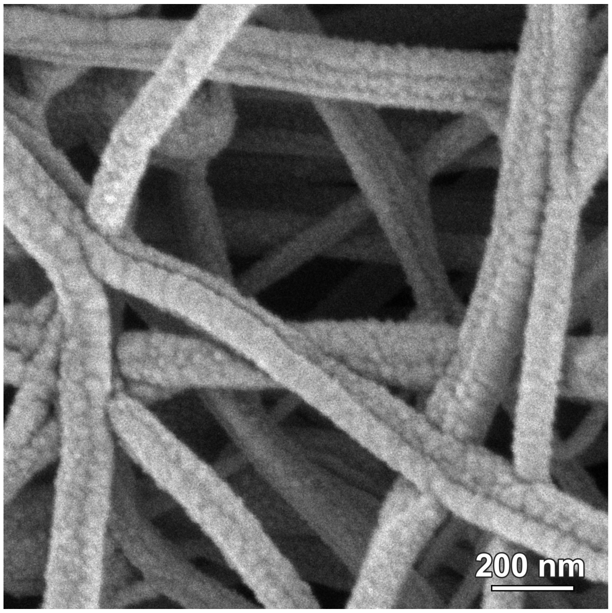

Supplementary Figure 6 | SEM image of Cu/PTFE.

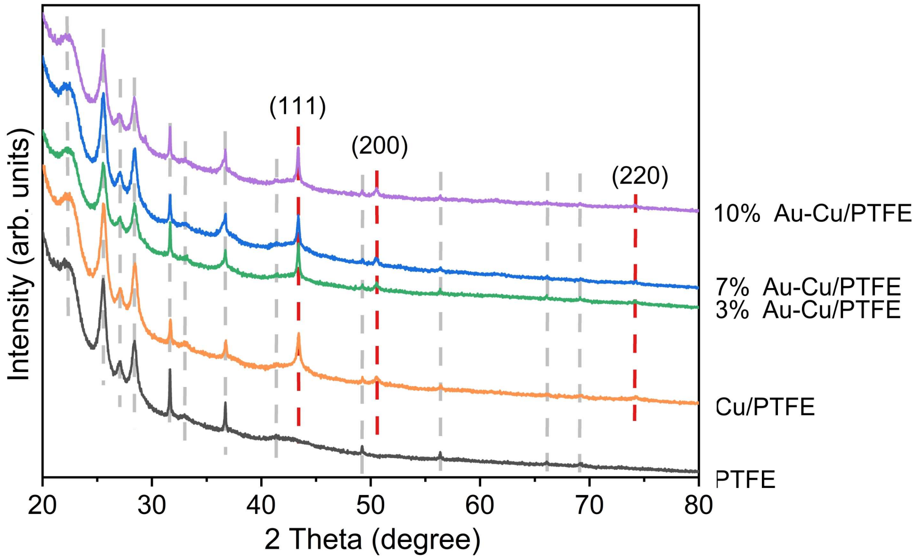

Supplementary Figure 7 | Powder X-ray diffraction (XRD) patterns of PTFE and different catalysts on PTFE. The peaks marked by gray dotted lines are derived from the PTFE substrates.

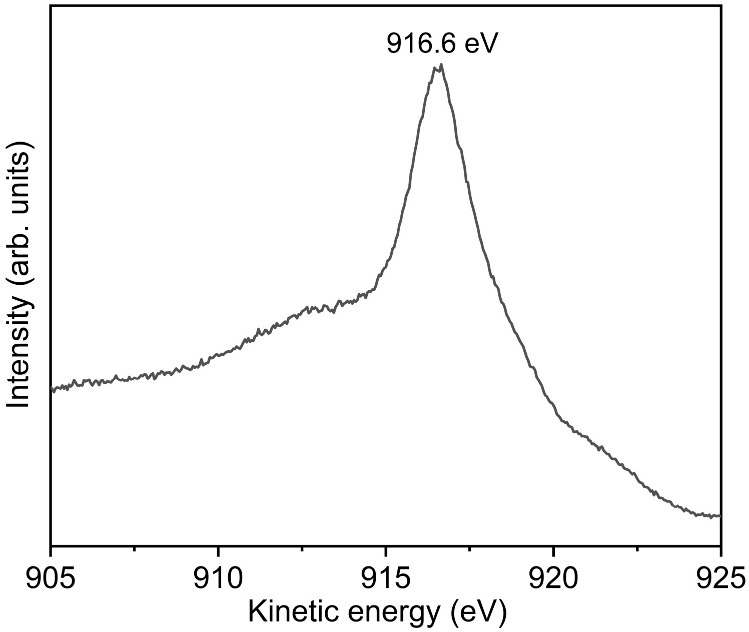

Supplementary Figure 8 | Cu LMM Auger spectrum of 7% Au-Cu/PTFE. The position of the peak in the Cu LMM Auger spectra (~916.6 eV) demonstrates the presence of Cu(I) in Au-Cu catalysts[1].

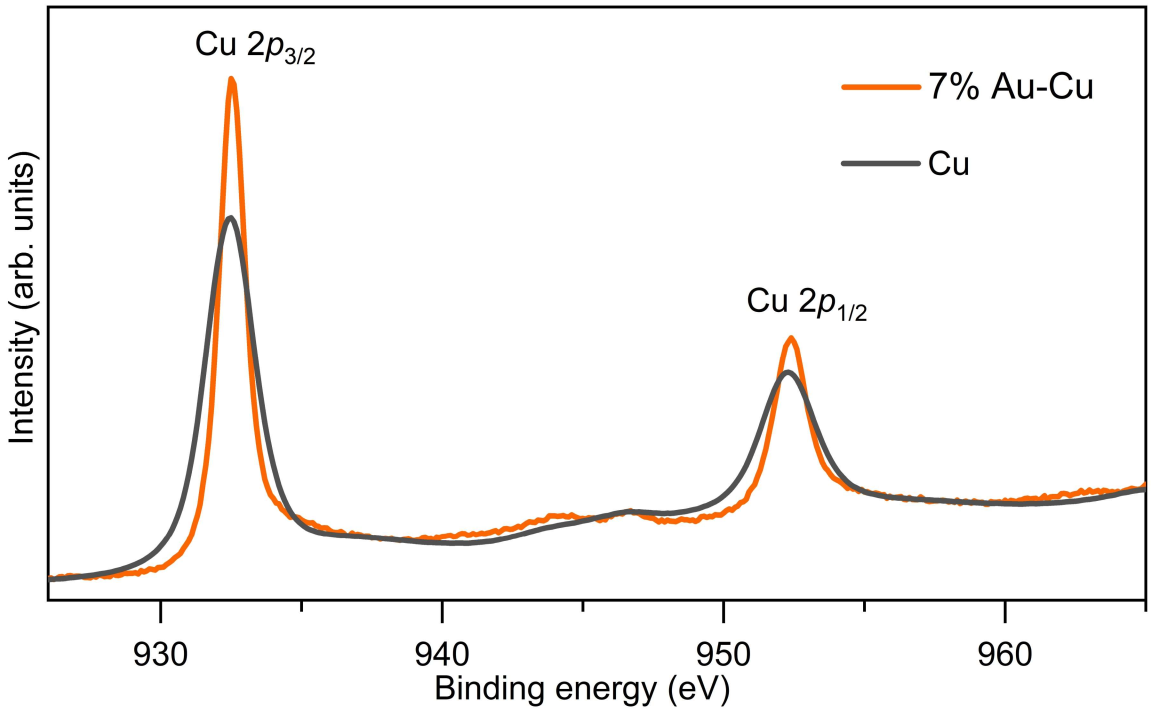

Supplementary Figure 9 | Comparison of Cu 2*p* spectra between 7% Au-Cu/PTFE and Cu/PTFE. Relative to Cu/PTFE, the Cu 2*p* spectrum for 7% Au-Cu/PTFE shifts to higher binding energy, indicating that the presence of a surface alloy in 7% Au-Cu/PTFE.

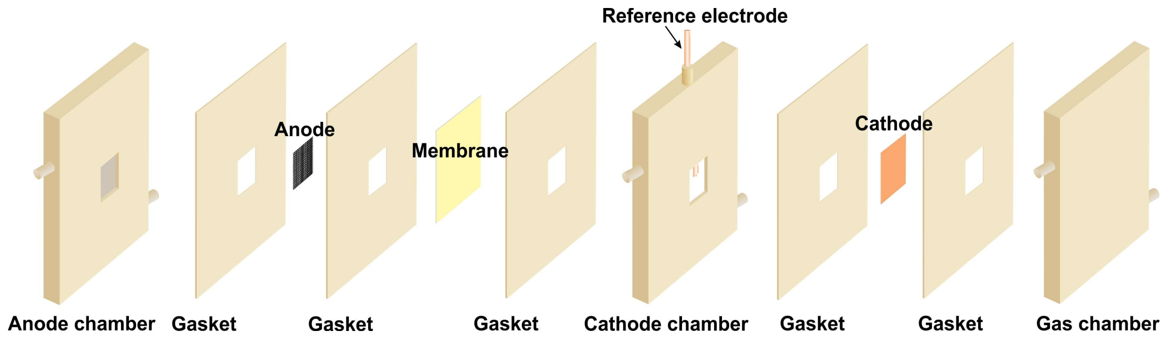

Supplementary Figure 10 | Schematic of flow cell. Chamber dimensions (anode chamber, cathode chamber, and gas chamber): 6 cm × 6 cm × 1.7 cm. Gasket dimensions: 6 cm × 6 cm × 0.1 cm. The square holes are 1 cm × 1 cm.

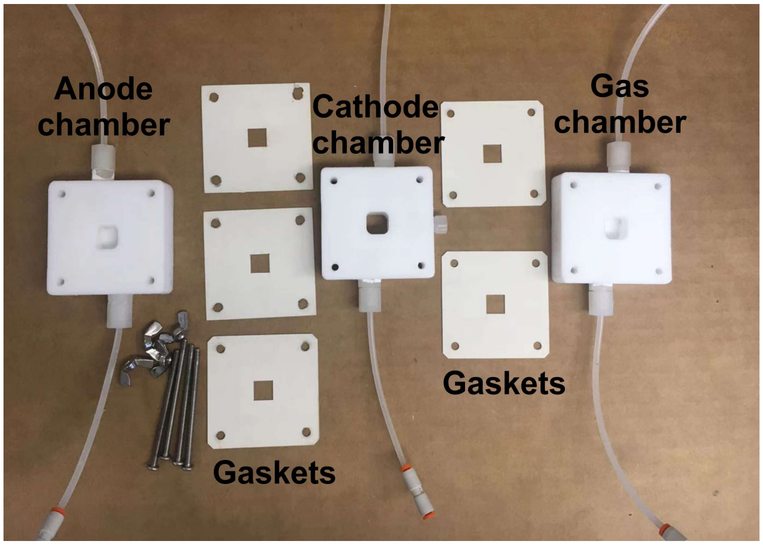

Supplementary Figure 11 | Photograph of flow cell components. Note: The anode, cathode, reference electrode, and membrane are not shown.

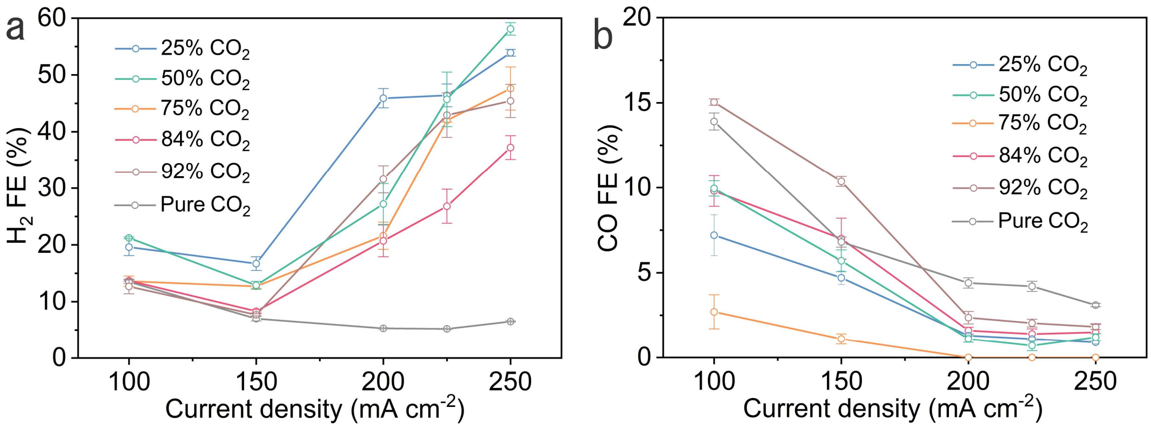

Supplementary Figure 12 | H_2 and CO FEs on 7% Au-Cu catalysts in CO_2RR at various CO_2 concentrations. **a**, H_2 FEs. **b**, CO FEs. Error bars represent the standard deviation based on three separate measurements.

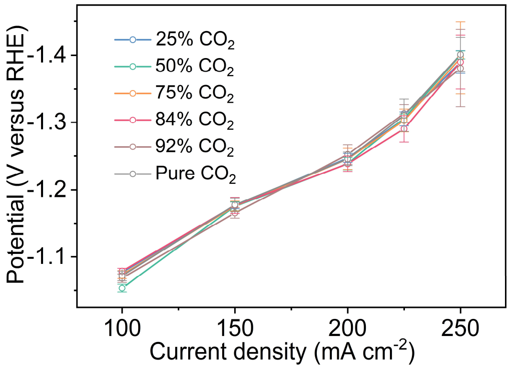

Supplementary Figure 13 | Total current densities versus potentials referenced to RHE on 7% Au-Cu catalysts at various CO_2 concentrations. Error bars represent the standard deviation based on three separate measurements.

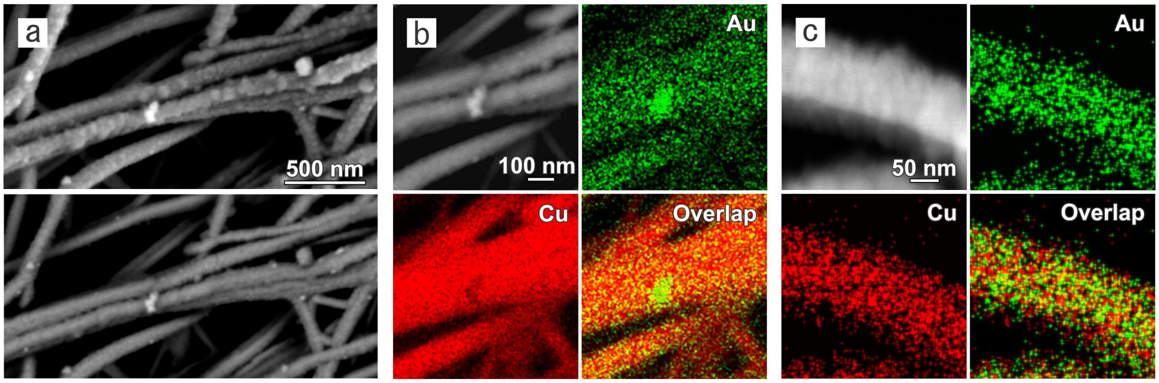

Supplementary Figure 14 | Structural and compositional analyses of 3% Au-Cu catalysts on PTFE. **a**, Low magnification secondary electron image (above) and the corresponding backscattered electron image (below) of the 3% Au-Cu/PTFE. **b,** Backscattered electron image and the corresponding EDX elemental mapping of Au and Cu for the 3% Au-Cu/PTFE. **c**, High magnification HAADF-STEM image and the corresponding elemental mapping of Au and Cu from a section of 3% Au-Cu/PTFE nanofibers.

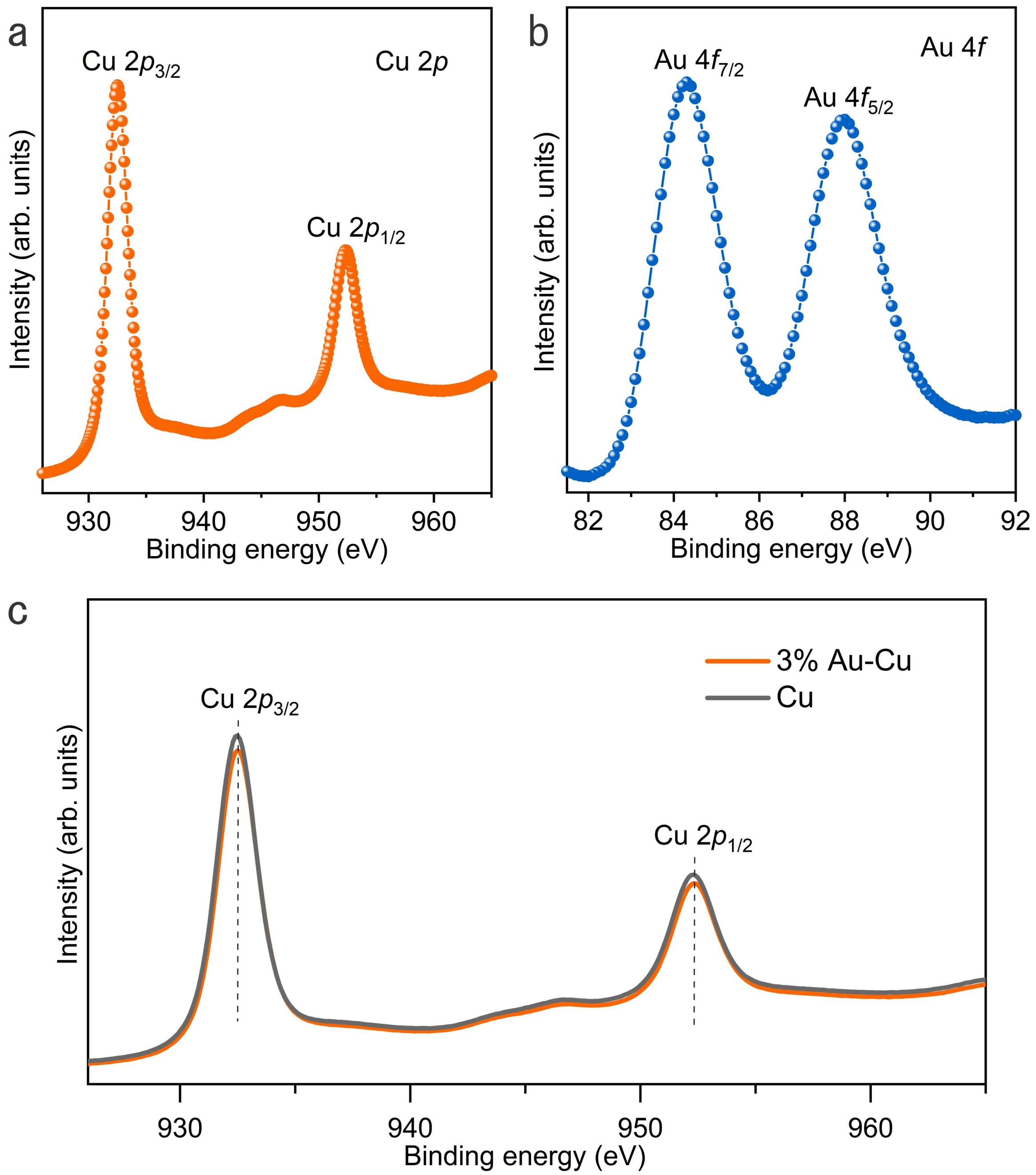

Supplementary Figure 15 | XPS analyses for 3% Au-Cu/PTFE. a, **b**, High-resolution XPS spectra of Cu 2*p* (**a**) and Au 4*f* (**b**) for 3% Au-Cu/PTFE. **c**, Comparison of Cu 2*p* spectra between 3% Au-Cu/PTFE and Cu/PTFE. Relative to Cu/PTFE, the Cu 2*p* spectrum for 3% Au-Cu/PTFE shifts to higher binding energy, indicating the presence of a surface alloy in 3% Au-Cu/PTFE.

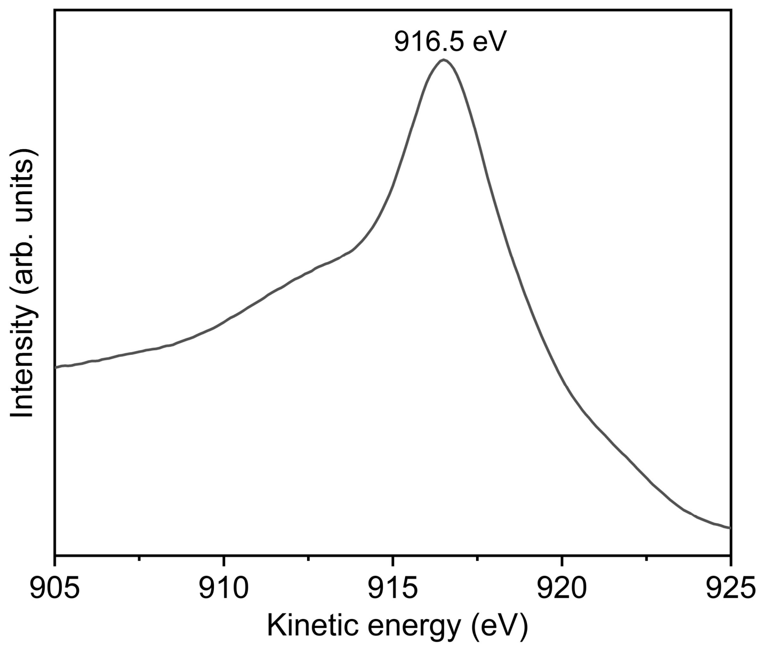

Supplementary Figure 16 | Cu LMM Auger spectrum of 3% Au-Cu/PTFE.

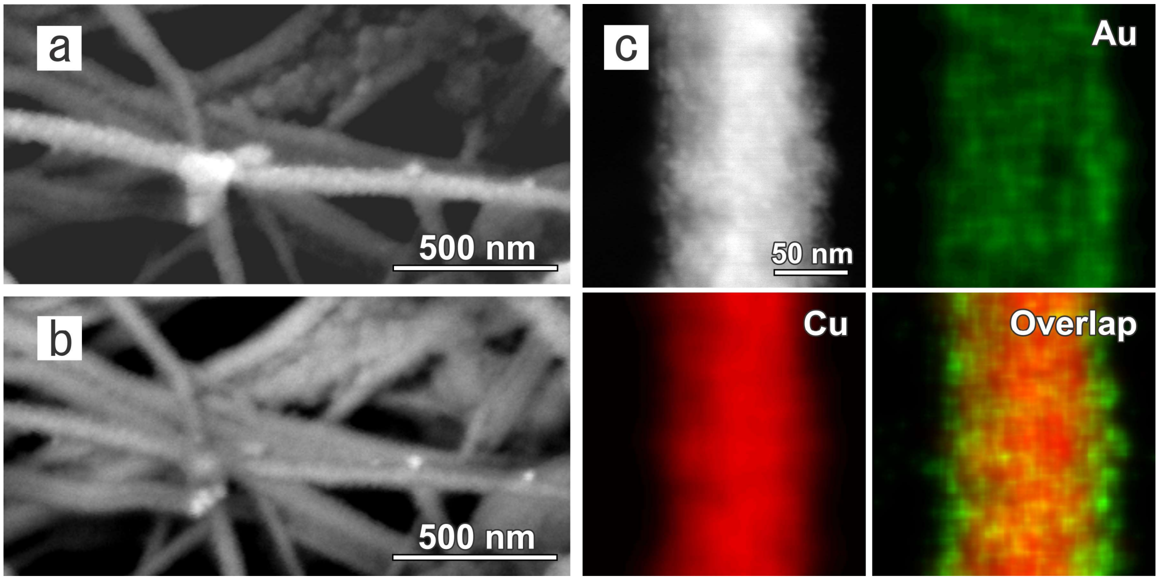

Supplementary Figure 17 | Structural and compositional analyses of 10% Au-Cu catalysts on PTFE. a,**b,** Low magnification secondary electron image (**a**) and the corresponding backscattered electron image of the 10% Au-Cu/PTFE, showing the dispersed Au nanoparticles (bright spots) (**b**). **c**, High magnification HAADF-STEM image and the corresponding elemental mapping of Au and Cu from a section of a 10% Au-Cu/PTFE nanofiber.

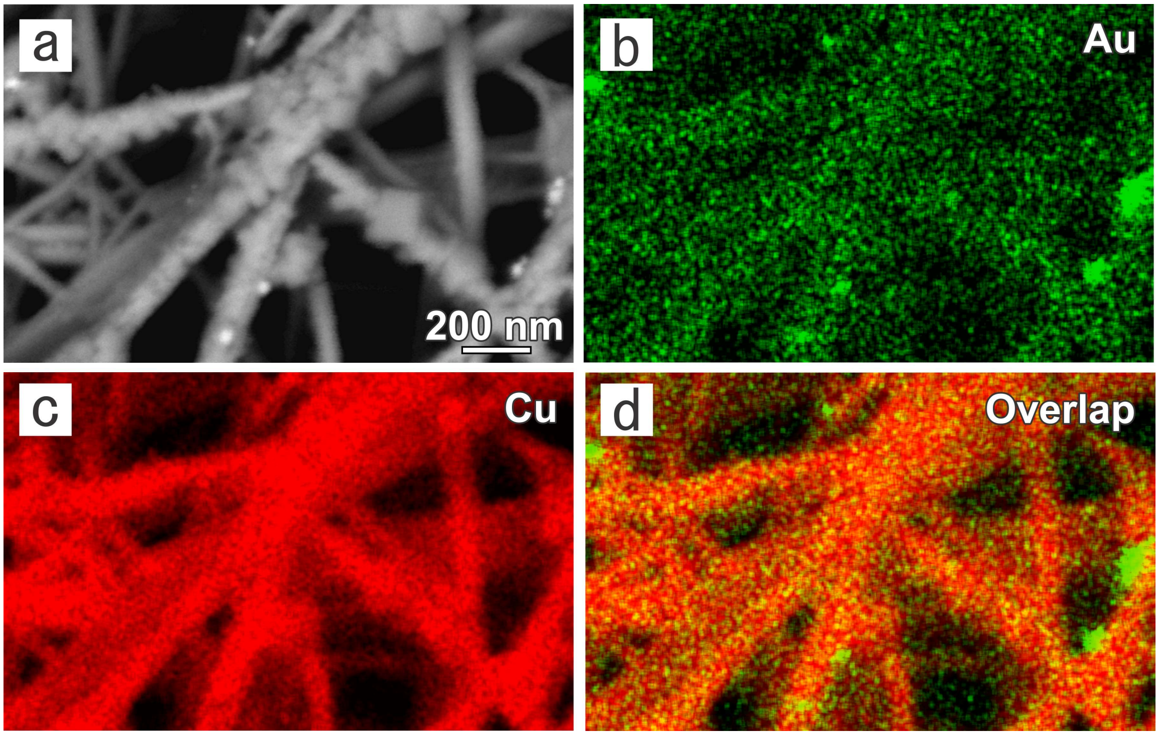

Supplementary Figure 18 | Structural and compositional analyses of 10% Au-Cu catalysts on PTFE. a, Backscattered electron image of the 10% Au-Cu/PTFE. **b-d,** The corresponding EDX elemental mapping of Au (**b**), Cu (**c**), and overlap of Au and Cu (**d**) for the 10% Au-Cu/PTFE. TEM/SEM characterization and the corresponding EDX elemental mapping of the 10% Au-Cu catalyst show that the elemental Cu and Au are dispersed evenly on PTFE nanofibers, together with some loosely distributed Au nanoparticles on the nanofibers, which is similar to 3% Au-Cu and 7% Au-Cu catalysts.

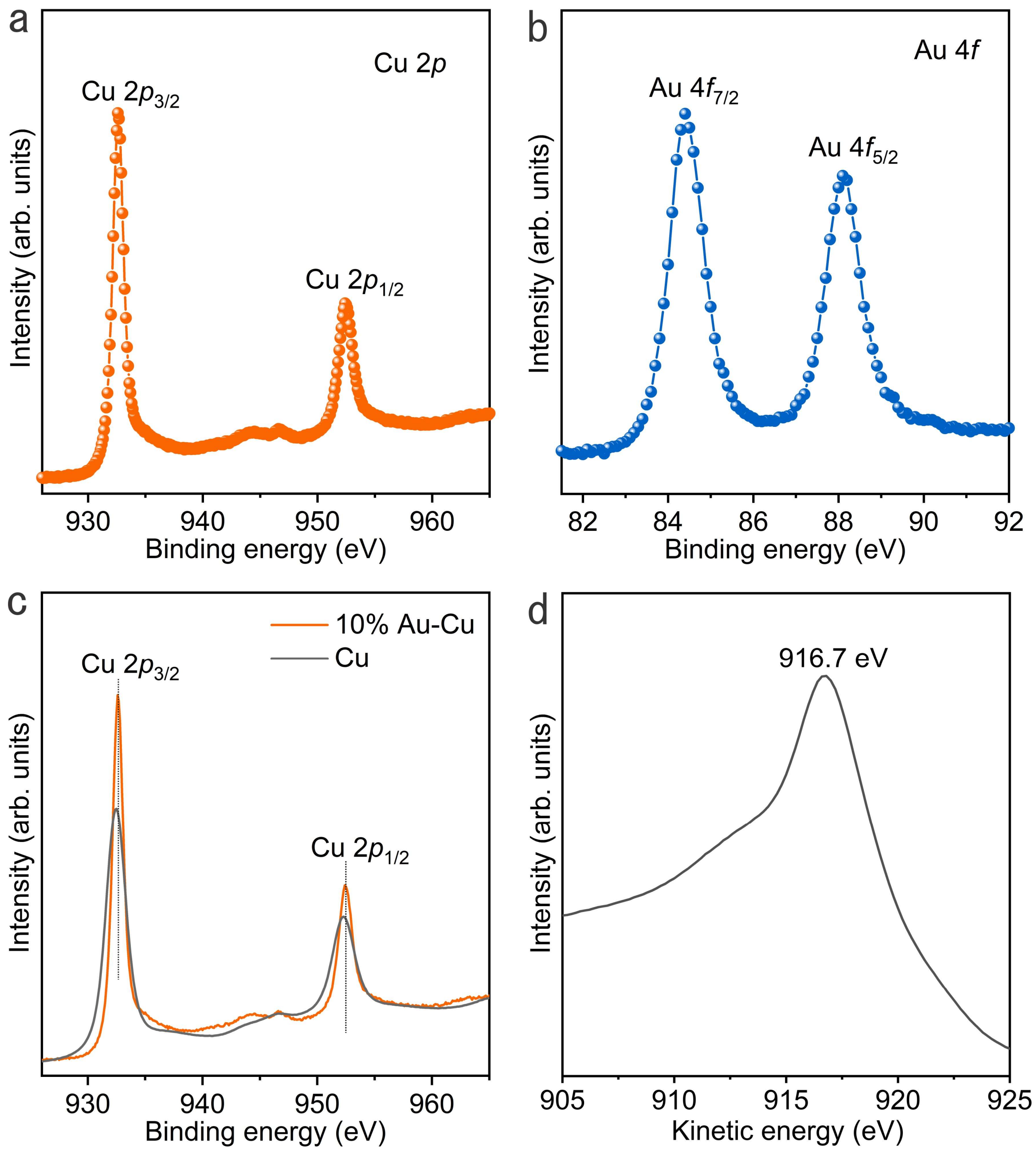

Supplementary Figure 19 | XPS analyses for 10% Au-Cu/PTFE. a, **b**, High-resolution XPS spectra of Cu 2*p* (**a**) and Au 4*f* (**b**) for 10% Au-Cu/PTFE. **c**, Comparison of Cu 2*p* spectra between 10% Au-Cu/PTFE and Cu/PTFE. **d**, Cu LMM Auger spectrum of 10% Au-Cu/PTFE. In Supplementary Fig. 19c, the Cu 2*p* spectrum for 10% Au-Cu/PTFE shifts to higher binding energy relative to Cu/PTFE, indicating the presence of a surface alloy in 10% Au-Cu/PTFE.

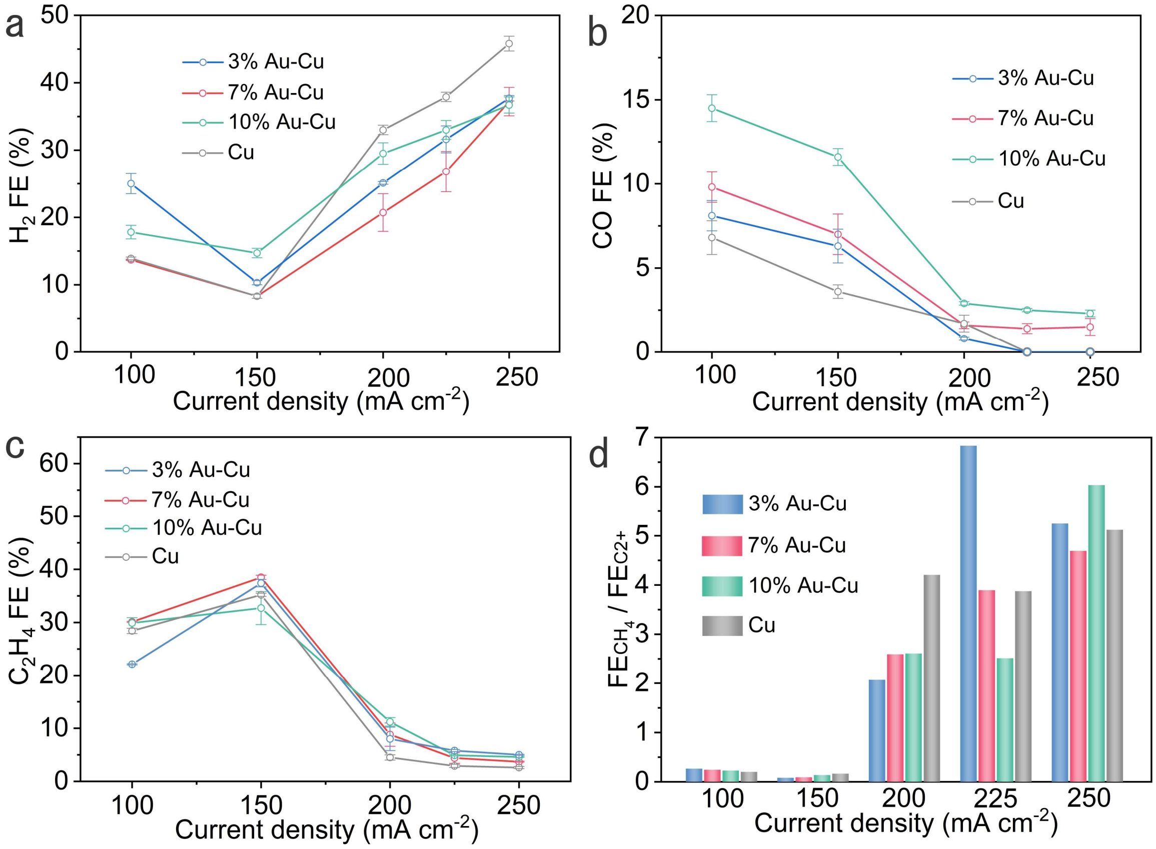

Supplementary Figure 20 | CO_2RR performance on different catalysts at 84% CO_2. a-c, FEs of H_2 (**a**), CO (**b**), and ethylene (**c**) on different catalysts. Error bars represent the standard deviation based on three separate measurements. **d**, Comparison of the ratios of FE_{CH_4} to FE_{C2+} on different catalysts.

At low reaction rates (⩽ 150 mA cm^{-2}), 3% Au-Cu and 10% Au-Cu catalysts exhibit higher H_2 FE than Cu catalysts. Relative to high reaction rates (200 – 250 mA cm^{-2}), the *CO coverage on catalyst surfaces is higher at low reaction rates (⩽ 150 mA cm^{-2}), which might lead to a lower *H coverage on the catalyst surface. Thus, higher H_2 FEs are observed on some Au-Cu catalysts vs. Cu catalysts, supported by DFT calculations (Fig. 1c).

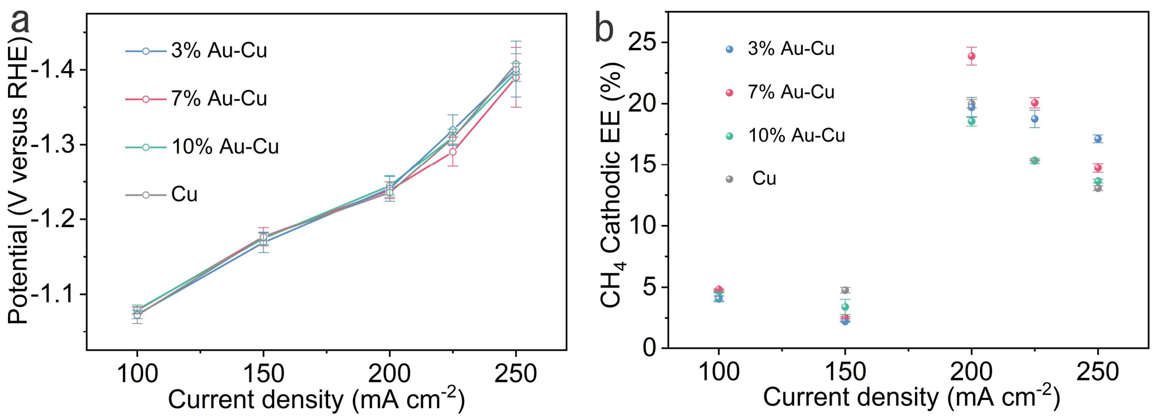

Supplementary Figure 21 | CO_2RR performance on different catalysts at 84% CO_2. a, Total current densities versus potentials referenced to RHE on different catalysts. **b**, CH_4 cathodic EEs on different catalysts at different current densities. Error bars represent the standard deviation based on three separate measurements.

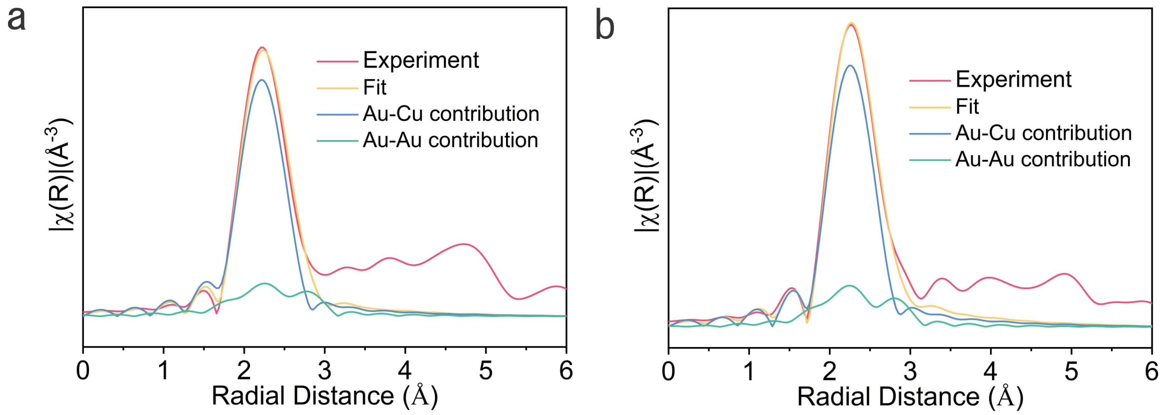

Supplementary Figure 22 | Fourier transforms of extended X-ray absorption fine structure (EXAFS) spectra for Au-Cu catalysts at the Au L3-edge. a, 3% Au-Cu catalysts. **b,** 7% Au-Cu catalysts.

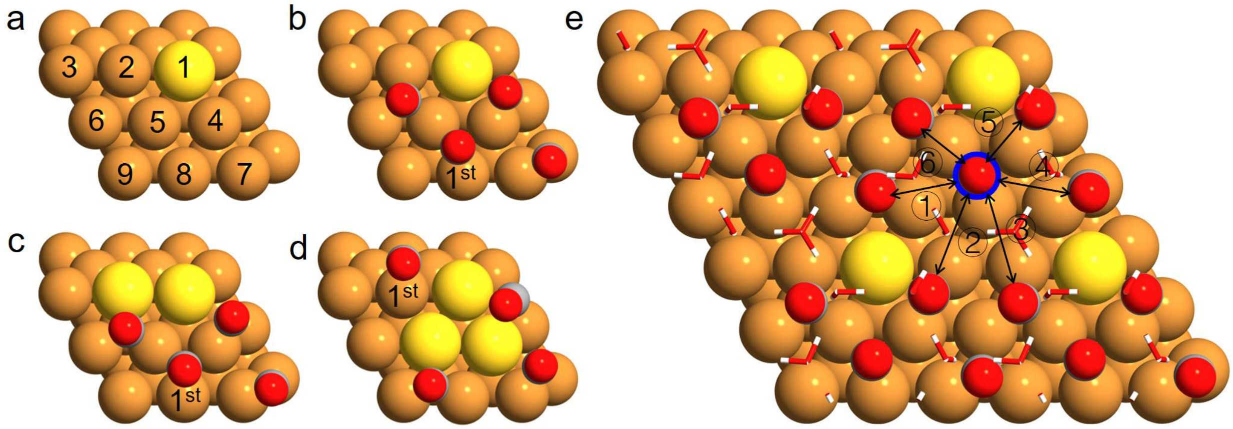

Supplementary Figure 23 | Computational workflow. a, Indexing surface atoms to determine adsorption sites for *CO adsorption (e.g., Au atop: 1; Cu atop: 2; Au-Cu bridge: 1-2; Cu-Cu bridge: 2-3; $AuCu_2$ fcc hollow: 125; $AuCu_2$ hcp hollow: 145; Cu_3 fcc hollow: 589; Cu_3 hcp hollow: 569, etc.). **b-d**, Top views of *CO intermediates under 4/9 ML on the surfaces of Au_1Cu_{35} (**b**), Au_2Cu_{34} (**c**), and Au_3Cu_{33} (**d**). 1st denotes the first adsorbed *CO in the sequence which is assumed to be the one that undergoes the *CO protonation and C-C coupling. **e**, Six different directions of C-C coupling considered in the present study.

Note: (1) We first screened for the most stable CO adsorption site (top, bridge, fcc, and hcp hollow sites from surface atom indices 1-9 in Supplementary Fig. 23a) on the (111) surfaces of Cu_{36}, Au_1Cu_{35}, Au_2Cu_{34}, and Au_3Cu_{33}, considering a single adsorbate and no solvent. We found out that the hcp hollow589 and fcc hollow569 are the most stable sites for the first CO adsorption with an adsorption energy of -0.96 eV. We then repeated this process to further determine the adsorption sites for the *CO intermediates under different coverages (2/9, 3/9, and 4/9 ML; 4/9ML scenarios are shown in Supplementary Fig. 23b-d) on various surfaces. (2) Based on the results from step 1, we used the chemical intuition garnered from this approach to distribute other adsorbates (*CHO, *OCCO, *OCCOH, and *H) on the slab under 2/9, 3/9, and 4/9 ML *CO coverage and added a monolayer of water molecules above the adsorbates. We assume the first adsorbed *CO in the sequence to be the most active species among the others, which undergoes the subsequent *CO protonation or C-C coupling. For example, under 4/9 ML *CO coverage on Au_1Cu_{35}, the 1st adsorbed *CO and its six nearest neighboring *CO were considered to participate in C-C coupling along six different directions, as shown in Supplementary Fig. 23e. We also considered different directions of *OCCO protonation.

Supplementary Table 1. Electrochemical CO_2-to-methane performance for studies having a total current density above 100 mA cm^{-2}.

Catalyst	$FE_{methane}$ (%)	$FE_{methane}/FE_{hydrogen}$	Cathodic EE (%) for methane	Reference
Au-Cu	56 ± 2	2.7	24 ± 1	This work
Sputtered Cu	48	1.7	20	*J. Am. Chem. Soc.* **2020**, 142, 3525
Cu	18	0.6	9	*Nat. Catal*. **2018**, 1, 111
Ag-Cu	15	0.8	8	*J. Am. Chem. Soc.* **2019**, 141, 8584
Cu-Pd	7.5	N/A	4	*J. Am. Chem. Soc.* **2017**, 139, 47
Cu_2S/Cu-V	2	0.2	N/A	*Nat. Catal*. **2018**, 1, 421

Supplementary Table 2. Free energy corrections for non-adsorbed and adsorbed species. E is the electronic energy determined by DFT calculations, ZPE, $\int C_p dT$, and $-TS$ represent the free energy corrections from zero-point energy, heat capacity, and entropy, respectively.

Species	ZPE	$\int C_p dT$	$-TS$	$G–E$
CO (g)	0.13	0.09	−0.61	−0.39
H_2 (g)	0.27	0.09	−0.40	−0.04
*H	0.17	0.01	−0.01	0.17
*CO	0.18	0.07	−0.11	0.14
*CHO	0.42	0.08	−0.12	0.38
*OCCOH	0.74	0.10	−0.17	0.67

Supplementary Table 3. Reaction free energies of *CO to *CHO (ΔG_{*CHO}) and C−C coupling (ΔG_{*OCCOH}) on Cu_{36}, Au_1Cu_{35}, Au_2Cu_{34}, and Au_3Cu_{33} surfaces under different *CO coverages.

Systems	*CO coverage	ΔG_{*CHO}	ΔG_{*OCCOH}
Cu_{36}	2/9	0.81	0.89
	3/9	0.79	0.86
	4/9	0.60	0.49
Au_1Cu_{35}	2/9	0.82	0.83
	3/9	0.72	0.68
	4/9	0.55	0.36
Au_2Cu_{34}	2/9	0.79	0.87
	3/9	0.69	0.54
	4/9	0.44	0.26
Au_3Cu_{33}	2/9	0.78	0.88
	3/9	0.53	0.39
	4/9	0.48	0.26

Supplementary Table 4. Liquid product FEs on 7% Au-Cu catalysts in CO_2RR at various CO_2 concentrations.

Catalyst	CO_2 concentration	J_{total} (mA cm^{-2})	$FE_{formate}$ (%)	$FE_{ethanol}$ (%)	$FE_{acetate}$ (%)	$FE_{n-propanol}$ (%)
7% Au-Cu	25%	100	2.1	22.2	2.6	1.6
		150	1.0	25.9	2.2	0
		200	2.1	10.8	2.3	0.4
		225	2.6	5.4	3.1	0.5
		250	3.2	5.5	2.4	0
	50%	100	3.9	18.1	2.0	3.1
		150	3.1	24.7	4.4	2.6
		200	5.8	7.8	3.8	2.2
		225	5.1	3.6	2.6	0
		250	5.0	0.9	0.9	0
	75%	100	13.9	7.3	1.7	2.2
		150	6.3	6.3	5.8	0.1
		200	5.5	6.8	1.4	0.1
		225	5.5	4.4	1.0	0.9
		250	5.6	4.2	1.0	0.6
	84%	100	7.3	8.6	1.4	2.9
		150	4.4	15.4	4.8	3.4
		200	1.5	7.5	4.4	0.8
		225	5.3	5.4	2.3	0.1
		250	3.5	2.6	1.2	0.2
	92%	100	4.7	16.4	2.0	3.2
		150	3.7	23.5	3.1	4.3
		200	4.2	16.0	7.1	1.3
		225	7.9	8.0	3.3	0.3
		250	7.5	4.2	3.8	0
	Pure CO_2	100	12.2	11.3	3.0	4.0
		150	5.8	20.1	3.5	4.9
		200	4.6	21.7	5.2	4.7
		225	4.7	23.3	5.8	4.3
		250	4.1	25.3	7.2	3.4

Supplementary Table 5. Liquid product FEs on different catalysts in CO_2RR under 84% CO_2.

Catalyst	J_{total} (mA cm^{-2})	$FE_{formate}$ (%)	$FE_{ethanol}$ (%)	$FE_{acetate}$ (%)	$FE_{n\text{-}propanol}$ (%)
3% Au-Cu	100	13.1	7.5	1.1	2.3
	150	5.1	15.7	5.5	2.2
	200	5.9	7.4	6.3	0.5
	225	6.9	0.7	0.1	0
	250	4.5	2.3	0.8	0
10% Au-Cu	100	7.0	6.4	2.0	2.1
	150	6.8	17.8	3.2	3.2
	200	3.1	3.9	1.4	0.1
	225	2.6	5.8	3.4	0.5
	250	3.5	0	0.9	0.1
Cu	100	6.0	16.6	1.4	4.1
	150	5.3	21.5	4.9	4.3
	200	7.2	5.5	0.6	0.5
	225	8.7	6.0	0	0.6
	250	4.7	3.4	0	0.4

Supplementary Table 6. Au L3-edge EXAFS fitting parameters for Au-Cu catalysts.

Samples	Shells	N	R (Å)	$\sigma^2 \times 10^3$ (Å^2)	ΔE_0 (eV)
3% Au-Cu	Au-Cu	8.5	2.59	7.27	1.6
	Au-Au	3.0	2.69	7.39	1.6
7% Au-Cu	Au-Cu	7.6	2.60	5.43	5.2
	Au-Au	4.1	2.68	8.48	5.2

N, coordination number; R, bonding distance; σ^2, Debye-Waller factor; ΔE_0, shift in adsorption edge energy.

Reference

1. Platzman, I., Brener, R., Haick, H. & Tannenbaum, R. Oxidation of polycrystalline copper thin films at ambient conditions. *J. Phys. Chem. C* **112**, 1101-1108 (2008).
